# Targeting endoplasmic reticulum stress‐induced lymphatic dysfunction for mitigating bisphosphonate‐related osteonecrosis

**DOI:** 10.1002/ctm2.70082

**Published:** 2024-11-09

**Authors:** Ziyue Qin, Hanyu Xie, Pengcheng Su, Zesheng Song, Rongyao Xu, Songsong Guo, Yu Fu, Ping Zhang, Hongbing Jiang

**Affiliations:** ^1^ Department of Oral and Maxillofacial Surgery, The Affiliated Hospital of Stomatology Nanjing Medical University Nanjing China; ^2^ State Key Laboratory Cultivation Base of Research, Prevention and Treatment for Oral Diseases Nanjing Medical University Nanjing Jiangsu China; ^3^ Jiangsu Province Engineering Research Center of Stomatological Translational Medicine Nanjing Medical University Nanjing Jiangsu China

**Keywords:** autophagy, bisphosphonates, endoplasmic reticulum stress, lymphatic drainage, osteonecrosis

## Abstract

**Background:**

Bisphosphonates (BPs) are the first‐line treatment to stop bone resorption in diseases, including osteoporosis, Paget's disease, multiple myeloma and bone metastases of cancer. However, BPs‐related osteonecrosis of the jaw (BRONJ), characterized by local inflammation and jawbone necrosis, is a severe intractable complication. The cumulative inflammatory burden often accompanies impaired lymphatic drainage, but its specific impact on BRONJ and the underlying mechanisms remain unclear.

**Methods:**

The mouse BRONJ model was established to assess the integrity and drainage function of lymphatic vessels by tissue clearing techniques, injected indocyanine green lymphatic clearance assay, flow cytometry analysis and histopathological staining. RNA sequencing, metabolome analysis, transmission electron microscopy and Western blotting were utilized to analyze the impacts of Zoledronate acid (ZA) on endoplasmic reticulum stress (ERS) and function of lymphatic endothelial cells (LECs). By constructing *Lyve1^creERT^; SIRT6^f/f^* and *Lyve1^creERT^; ATG5^f/f^* mice, we evaluated the role of ERS‐induced LECs apoptosis in the progression of BRONJ. Additionally, we developed a nanoparticle‐loaded ZA and rapamycin (ZDPR) to enhance autophagy and evaluated its potential in mitigating BRONJ.

**Results:**

The mouse BRONJ model displayed impaired lymphatic drainage, accompanied by significant local inflammation and bone necrosis. The prolonged stimulation of ZA resulted in the extension of ERS and the inhibition of autophagy in LECs, ultimately leading to apoptosis. Mechanistically, ZA activated XBP1s through the NAD^+^/SIRT6 pathway, initiating ERS‐induced apoptosis in LECs. The conditional knockout mouse models demonstrated that the deletion of *SIRT6* or *ATG5* significantly worsened lymphatic drainage and inflammatory infiltration in BRONJ. Additionally, the innovative nanoparticle ZDPR alleviated ERS‐apoptosis in LECs and enhanced lymphatic function, facilitating inflammation resolution.

**Conclusion:**

Our study has elucidated the role of the NAD^+^/SIRT6/XBP1s pathway in ERS‐induced apoptosis in ZA‐treated LECs, and further confirmed the therapeutic potential of ZDPR in restoring endothelial function and improving lymphatic drainage, thereby effectively mitigating BRONJ.

**Key points:**

- Bisphosphonate‐induced lymphatic drainage impairment exacerbates bone necrosis.
- Zoledronate acid triggers endoplasmic reticulum stress and apoptosis in lymphatic endothelial cells via the NAD+/SIRT6/XBP1s pathway.
- Novel nanoparticle‐loaded Zoledronate acid and rapamycin enhances autophagy, restores lymphatic function, and mitigates bisphosphonates‐related osteonecrosis of the jaw progression.

## Graphical abstract

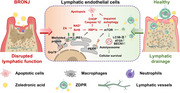

## 1 INTRODUCTION

Bisphosphonates (BPs), including Zoledronate acid (ZA), Alendronic acid etc., are the cornerstone of therapy for bone diseases such as Paget's disease, bone metastases and osteoporosis.^1^, ^2^, ^3^, ^4^ BPs inhibit bone resorption by binding to the mineralized bone surface and disrupting osteoclast activity.^5^ However, bisphosphonate‐related osteonecrosis of the jaw (BRONJ), a rare but intractable disease characterized by jawbone necrosis, remains a severe adverse effect of BPs. It often occurs post‐dental surgery or spontaneously in patients with a history of bisphosphonate use.^6^ BRONJ presents a significant clinical challenge with symptoms such as gingival dehiscence, necrotic bone exposure and infection.^7^ These issues collectively worsen the quality of life due to extensive soft and hard tissue defects in the oral and maxillofacial regions.^8^ Increasing evidence suggests that immune dysregulation and persistent inflammation are key pathological features of BRONJ,^9^ but the specific mechanisms remain unclear. The unique anatomical and microbiological characteristics of the jaw, including exposure to oral bacteria and frequent microtrauma, may contribute to exacerbating the inflammatory response and worsening the inflammatory microenvironment. Therefore, lymphatic drainage for alleviating inflammation and maintaining microenvironmental homeostasis in bone tissue may offer a new approach to the prevention and treatment of BRONJ.

The lymphatic system plays a crucial role in maintaining fluid homeostasis, facilitating waste clearance and orchestrating immune responses, particularly during inflammatory processes.^10^ The presence and functional significance of the lymphatic system in tissues such as the brain and bones, previously believed to be absent, have been confirmed by recent studies for tissue homeostasis and immune surveillance.^11^, ^12^ Lymphatic vessels are critical components of the bone microenvironment, and impaired lymphangiogenesis in disease states can hinder both hematopoietic and bone regeneration.^13^ By capturing soluble antigens from wound sites and transporting them to draining lymph nodes, lymphatic vessels create an immune‐regulatory niche,^14^ which is an underappreciated interface in the relief of inflammation in tooth extraction sockets (TES). As observed in BRONJ models, increased inflammation and delayed healing are associated with suppressed lymphangiogenesis.^15^ Besides, ZA induces oxidative stress and altered cellular oxidative metabolism in endothelial cells, leading to autophagic cell death.^16^, ^17^ This process increases the expression of inflammatory marker proteins, ultimately resulting in endothelial dysfunction. However, the impact of ZA on lymphatic endothelial cells (LECs) and its role in the development of lymphatic dysfunction during the BRONJ remains poorly elucidated.

Previous research has demonstrated that ZA can trigger endoplasmic reticulum stress (ERS)‐mediated inflammation in BRONJ, and the inhibition of this pathway could potentially ameliorate ZA‐induced BRONJ in vivo.^18^ ERS is a cellular response to alleviating stress caused by the abnormal accumulation of misfolded proteins within the endoplasmic reticulum (ER).^19^ Simultaneously, it induces autophagy, a process that selectively targets and degrades unfolded or misfolded proteins in the ER by sequestering them within autophagosomes and transporting them to lysosomes for degradation.^20^ The process involves the activation of ER transmembrane signalling molecules such as Protein kinase R‐like ER kinase (PERK) and inositol‐requiring enzyme 1 (IRE1).^21^ IRE1 then facilitates the mRNA splicing of the transcription factor X‐box binding protein 1 (XBP‐1), a major regulator of chaperones.^22^ Meanwhile, prolonged ER stress triggers a pro‐apoptotic pathway involving CCAAT/‐enhancer‐binding protein homologous protein (CHOP).^23^ Recent research has clarified the function of sirtuin 6 (SIRT6) in regulating the ERS response in endothelial cells.^24^ SIRT6, a NAD^+^‐dependent deacetylase, is known for its role in DNA repair, inflammation and metabolism.^25^ Under ERS conditions, SIRT6 deacetylates XBP1s, reducing its stability and promoting its degradation through the ubiquitin‐proteasome system.^26^ The deletion of SIRT6 has been demonstrated to exacerbate ERS, ultimately inducing apoptosis and metabolic dysregulation in endothelial cells^27^ and various other tissues.^28^ Despite these advancements, the interaction between ERS and SIRT6 is still not fully explored.

Here, we demonstrate that ZA treatment disrupts lymphatic drainage, thereby exacerbating the local accumulation of inflammation in BRONJ. Furthermore, ZA activated XBP1s through the NAD^+^/SIRT6 pathway, leading to LEC apoptosis induced by ERS. Using two conditional knockout mouse models, we further validated that ERS‐induced apoptosis of LECs impaired lymphatic vessel integrity and drainage function. Additionally, we developed nanoparticles loaded with rapamycin (RAPA) and ZA to not only inhibit osteoclast function but also reduce ERS‐induced apoptosis in LECs by enhancing autophagy, thereby restoring inflammatory homeostasis in the jawbone.

## 2 RESULTS

### 2.1 The BRONJ mouse model shows pronounced inflammatory accumulation, concomitant with compromised lymphatic drainage

**FIGURE 1:**
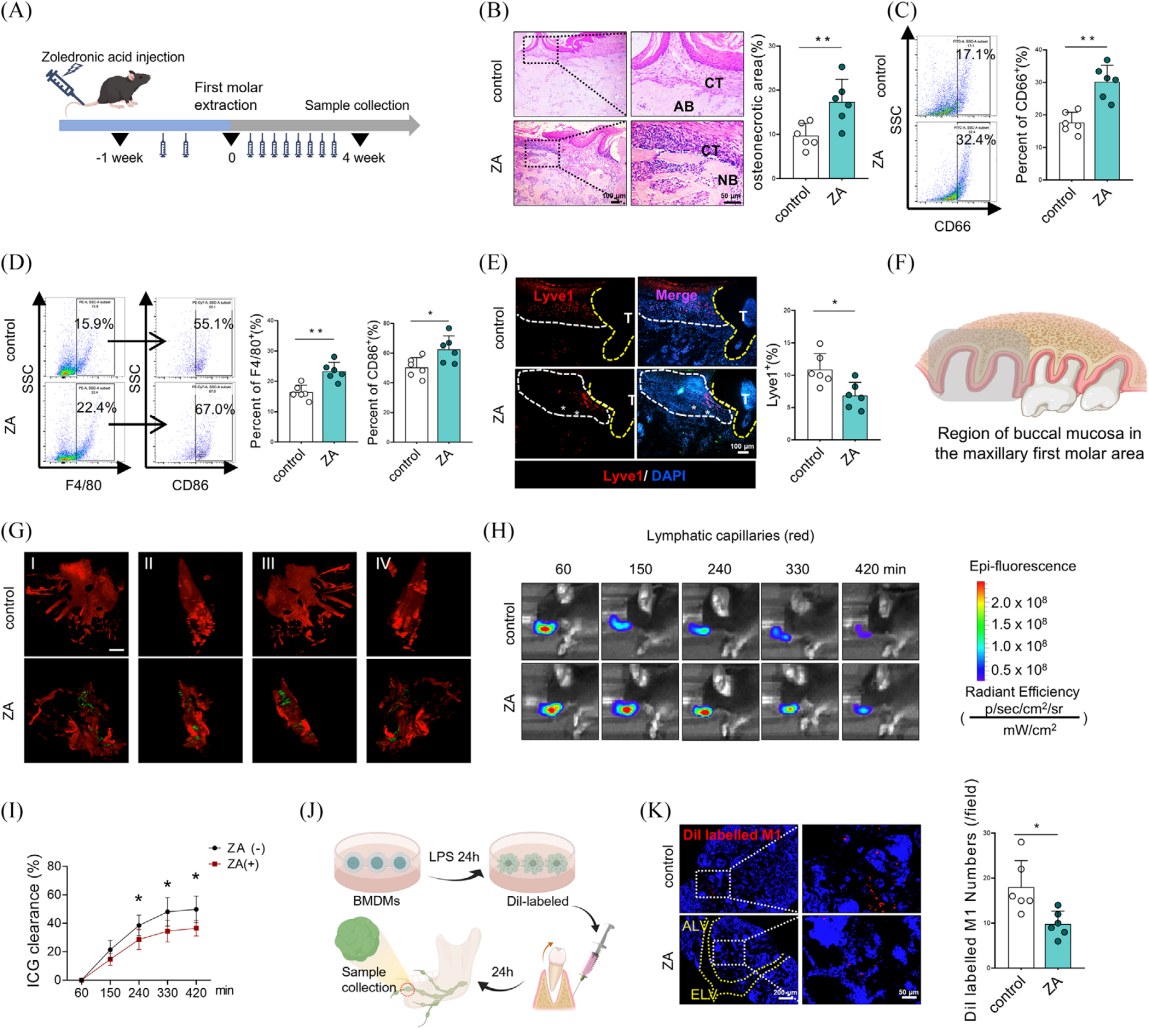
Bisphosphonates‐related osteonecrosis of the jaw (BRONJ) mice exhibit an accumulation of inflammatory cells in the tooth extraction socket, accompanied by obstruction of lymphatic reflux. (A) Schematic representation of the BRONJ model. (B) Representative H&E images of the tooth extraction socket at 4 weeks post‐extraction. AB, alveolar bone; CT, connective tissue; NB, necrotic bone. (C) Flow cytometric analysis of neutrophils in the tooth extraction sockets. (D) Flow cytometric analysis of M1 macrophages in the tooth extraction sockets. (E) Fluorescence staining and quantitative analysis of Lyve1^+^ cells in the tooth extraction wounds. T, tooth. (F) Schematic diagram of the target area for maxillary tissue clearing. (G) Imaging of local lymphatic vessels and macrophage distribution after maxillary tissue clearing. (H) Near‐infrared imaging showing clearance rates of indocyanine green (ICG) by lymphatic vessels in maxillary mucosa. (I) Quantitative analysis of Figure H. (J) Schematic diagram for the lymphatic drainage function of M1 macrophages. (K) Fluorescence staining and quantitative analysis of M1 macrophages in submandibular lymph nodes. ALV, afferent lymphatic vessels; ELV, efferent lymphatic vessels. A total of six subjects were analyzed. The results are presented as the mean ± standard deviation. *p < .05; ** *p* < .01; *** *p* < .001.

To investigate the potential pathogenic mechanisms of BRONJ, tail vein injection of ZA followed by the extraction of the maxillary first molars were conducted to establish a BRONJ disease model (Figure 1A). Relative to the control group, the ZA‐treated group demonstrated increased inflammatory cells and enlarged areas of necrotic bone in the TES (Figure 1B). The micro‐computed tomography (micro‐CT) analysis showed a significant reduction in bone volume/total volume (BV/TV) and bone mineral density (BMD) in the ZA‐treated group (Figure S1A). Notably, the ZA‐treated group exhibited a significant increase in neutrophil count (Figure 1C and Figure S1C), as well as the polarization of pro‐inflammatory M1 macrophages (Figure 1D and Figure S1B), thereby exacerbating inflammatory accumulation in the TES.

Considering the central role of the lymphatic system and prolonged inflammation in the pathogenesis of BRONJ.^29^ We first analyzed the distribution and quantitative changes of lymphatic vessels in BRONJ and found a notable decrease of Lyve1^+^ LECs compared to the control group (Figure 1E). Secondly, we conducted tissue clearing on maxillary bones and immunolabelled lymphatic vessels and macrophages (Figure 1F,G), and clearly observed the distribution and quantity of lymphatic vessels and macrophages around TES from four perspectives: buccal, lingual, mesial and distal through three‐dimensional reconstructions. In the control group, abundant lymphatic vessels were observed surrounding the TES, with a relatively low level of macrophage retention within the tissue. Conversely, in the ZA treatment group, irregularly shaped lymphatic vessels were noted in the tissues surrounding the TES, accompanied by a significant retention of macrophages (Figure 1G). To further analyze the function of lymphatic vessels, we injected indocyanine green (ICG) into the buccal region of mice and monitored the intensity of ICG signals at different time points. The clearance rate of ICG was assessed and thereby the drainage function of the periodontal lymphatic system was evaluated. A significant decrease in ICG clearance rate within the buccal periodontal tissues was observed in the ZA‐treated group (Figure 1H,I). Furthermore, Dil‐labeled M1 macrophages were administered into the submucosa of the buccal region around the TES. After 24 h, the submandibular lymph nodes were collected to analyze the impact of ZA on the drainage of M1 macrophages (Figure 1J). The results revealed a significant reduction in the quantity of Dil‐labeled M1 macrophages within the lymph nodes, indicating impaired lymphatic drainage function (Figure 1K). Together, an accumulation of inflammatory cells, reduced LECs and impaired lymphatic drainage are exhibited in the progression of BRONJ.

### 2.2 The NAD^+^/SIRT6 pathway alleviates ERS‐induced apoptosis in ZA‐treated LECs

**FIGURE 2:**
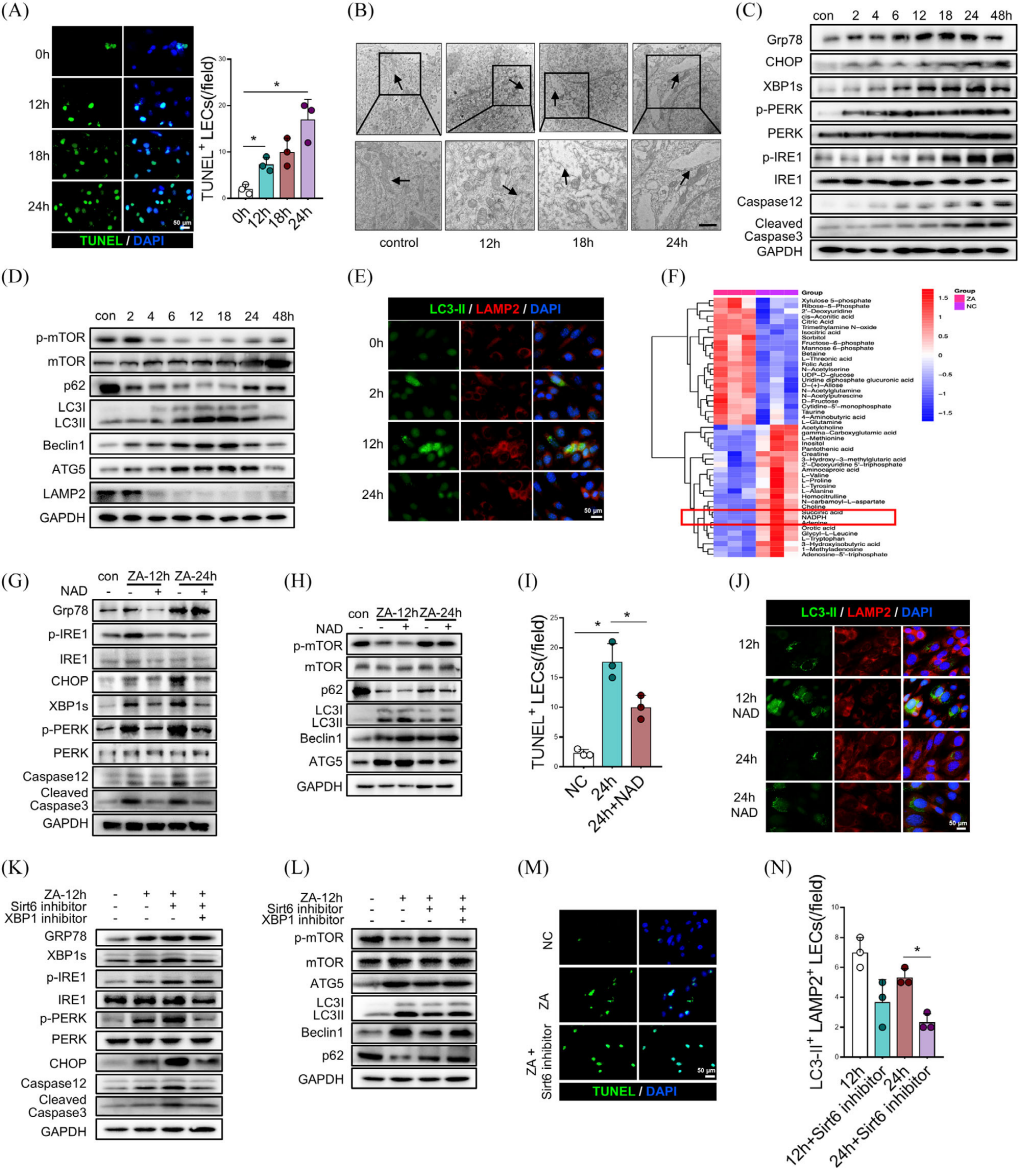
Zoledronate acid (ZA) induces endoplasmic reticulum stress (ERS)‐apoptosis in lymphatic endothelial cells through the NAD^+^/SIRT6 pathway. (A) TUNEL staining of lymphatic endothelial cells (LECs) at different time points after ZA treatment. (B) Transmission electron microscopy of LECs at different time points after ZA treatment. (C) Western blot analysis of the expression of ERS‐related proteins and (D) autophagy‐related proteins in LECs at different time points after ZA treatment. (E) Immunofluorescence staining of autolysosomes in LECs at different time points after ZA treatment. (F) Metabolomic analysis of ZA‐treated LECs. (G, H) Western blot analysis of ERS and autophagy‐related protein expression in LECs treated with NAD^+^ and ZA. (I) Quantitative analysis of the proportions of apoptotic LECs using TUNEL staining. (J) Immunofluorescence staining to detect the levels of autolysosomes in LECs. (K, L) Western blot analysis of ERS and autophagy‐related protein expression in LECs treated with SIRT6 or XBP1 inhibitors. Immunofluorescence detection of apoptotic LEC proportions (M), and expression levels of autolysosomes (N). A total of three subjects were analyzed. The results are presented as the mean ± standard deviation. *p < .05; **p < .01.

In the pathogenesis of BRONJ, we initially discovered lymphatic reflux dysfunction accompanied by a decrease in LECs quantity, yet the specific mechanistic remains unclear. We first subjected ZA‐treated LECs to RNA‐sequencing (RNA‐seq), revealing ZA‐induced LECs apoptosis (Figure S2A). Subsequently, we analyzed the pro‐apoptotic effect of ZA at different time points and observed that the LECs apoptosis rate was gradually increased with the prolongation of ZA stimulation within 24 h (Figure 2A). To further examine the mechanisms of apoptosis, we analyzed RNA‐seq results and found significant enrichment of ERS‐related pathways in the ZA‐treated group (Figure S2A). Subsequently, transmission electron microscopy revealed morphological changes in ER under ZA stimulation, transitioning from a compact state to a sparse and expanded morphology (Figure 2B). The expression levels of ERS markers (GRP78, XBP1s, p‐PERK/PERK and p‐IRE/IRE) also exhibited a progressive increase with prolonged exposure to ZA, reaching their peak at 24 h (Figure 2C and Figure S2B). Among these, the expression of the ER chaperone GRP78 and transcription factor XBP1s exhibited a slight decrease after 48 h, while pro‐apoptotic protein CHOP, ERS‐specific apoptosis molecule Caspase‐12 and apoptotic effector Cleaved Caspase3 gradually increased, reaching a peak at 48 h post‐treatment (Figure 2C). These results suggested that the activation of ERS response occurred within 24 h after ZA treatment, followed by an overload of ERS which triggered apoptosis. Furthermore, moderate ERS can accurately detect slowly or incompletely folded proteins and initiate ubiquitination and protein degradation through proteasome or autophagy‐mediated pathways. We, therefore, examined the expression of autophagy‐related proteins (p‐mTOR/mTOR, LC3B, p62, Beclin1, ATG5 and LAMP2) in ERS‐induced apoptosis. The results revealed a significant time‐dependent increase in autophagic response during treatment, reaching its peak at 24 h and subsequently declining by 48 h (Figure 2D and Figure S2C). Immunofluorescence results also confirmed a peak in the levels of LC3‐II and LAMP2 double‐positive autolysosomes at 12 h (Figure 2E and Figure S2D).

Besides maintaining protein homeostasis, ER is also a major site for lipid metabolism, participating in maintaining metabolic homeostasis.^23^ Thus, imbalanced ER function after ZA treatment likely leads to metabolic disruption, triggering apoptosis. Combined with metabolomic results, we found that bisphosphonates mainly activate the tricarboxylic acid cycle (TCA cycle) within cells, while significantly reducing cellular NADPH content (Figure 2F). To further elucidate the relationship between the TCA cycle and ER stress‐induced apoptosis, we treated LECs with the combination of ZA and NAD^+^, the important redox coenzyme of the TCA cycle. Compared to ZA treatment alone, combined treatment with NAD^+^ and ZA significantly alleviated ERS and consequent apoptosis (Figure 2G, I and Figures S2F and S3A). Additionally, autophagy‐related pathways were activated after NAD^+^ co‐treatment, evidenced by the expression of autophagy‐related proteins (Figure 2H and Figure S2E) and an increase in LC3‐II and LAMP2 double‐positive autolysosomes (Figure 2J and Figure S3B). SIRT6, a NAD^+^‐dependent histone deacetylase, has previously been found to regulate endothelial ER stress and suppress apoptosis.^24^ Additionally, SIRT6 could respond to ERS by regulating the degradation of XBP1 protein via the proteasomal pathway to inhibit apoptosis.^26^ These findings prompted us to investigate whether NAD^+^ may regulate XBP1 activity through SIRT6 to alleviate ERS. We used the SIRT6 inhibitor OSS_128167 at an optimized concentration of 100 µM for 12 h, as determined by CCK8 assays, which showed that concentrations above 200 µM reduced cell viability (Figure S3D). This concentration effectively inhibited SIRT6 expression by 50–60%, as confirmed by Western blot (Figure S3C). We then interfered with SIRT6 in ZA‐treated LECs and observed that SIRT6 inhibition exacerbated ZA‐induced ERS (Figure 2K and Figure S3E) and down regulated autophagy levels (Figure 2L,N and Figure S3H,I), resulting in a significant increase in apoptotic cells (Figure 2M Figure S3J). However, adding an XBP1s inhibitor on top of SIRT6 inhibition mitigated ERS‐induced apoptosis. To further evaluate SIRT6's therapeutic potential, we treated ZA‐induced BRONJ mice with the SIRT6 activator MDL‐800. Histological analysis of the tooth extraction sites showed a reduction in necrotic bone area, indicating that SIRT6 activation alleviates ZA‐induced bone pathology (Figure S3F,G). Taken together, our findings suggest that ZA exerts inhibitory effects on SIRT6 function through its impact on NAD^+^ metabolism, leading to upregulation of XBP1s and subsequent induction of ERS‐related apoptosis in LECs.

### 2.3 *Lyve1 ^creERT^*; *SIRT6^fl/fl^* mice display aggravated inflammation accumulation and lymphatic dysfunction

**FIGURE 3:**
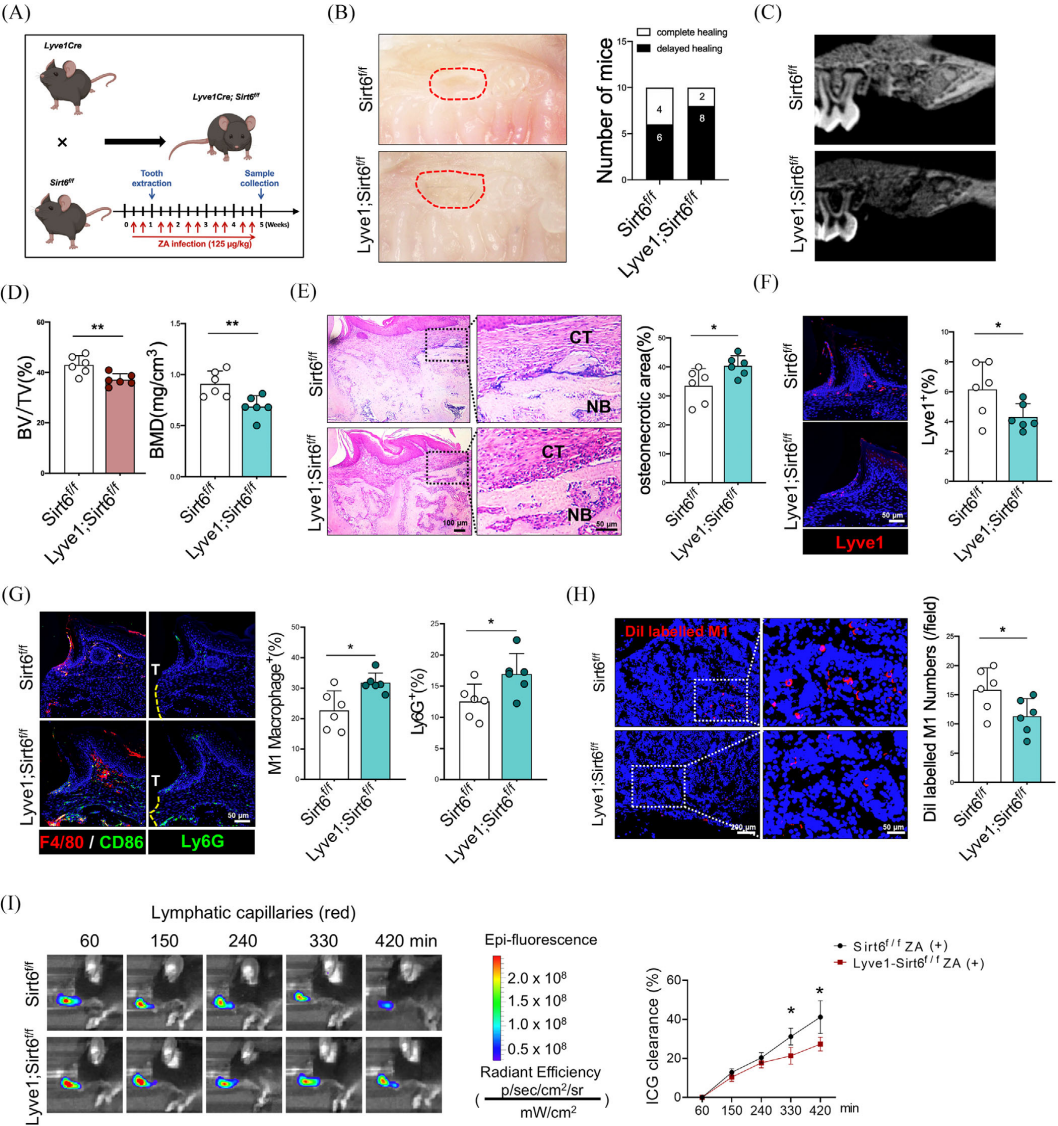
*Lyve1^creERT^; SIRT6^fl/fl^* mice exhibit exacerbated BRONJ phenotype and lymphatic reflux dysfunction. (A) Schematic representation of the construction of the conditional knockout animal model. (B) Gross morphology of the tooth extraction sockets in mice. (C) Micro‐computed tomography (micro‐CT) analysis of tooth extraction sockets healing. (D) The trabecular bone volume/tissue volume (BV/TV) and bone mineral density (BMD) of tooth extraction sockets were calculated by micro‐CT analysis. (E) HE staining to observe histological morphology of tooth extraction sockets. CT, connective tissue; NB, necrotic bone. (F) Immunofluorescence staining to observe lymphatic vessels labelled with Lyve1. (G) Immunofluorescence staining to observe the infiltration of neutrophils and macrophages in mucosal tissue of tooth extraction sites. T, tooth. (H) Immunofluorescence staining to observe macrophage drainage into submandibular lymph nodes. (I) Lymphatic reflux functional experiments in the region of maxillary tissue. A total of six subjects were analyzed. The results are presented as the mean ± standard deviation. *p < .05; **p < .01.

To further elucidate the role of SIRT6 in ERS‐induced apoptosis, we generated *Lyve1* *^creERT^; SIRT6^fl/fl^* mice (cKO) to specifically knockout SIRT6 in LECs and established BRONJ model (Figure 3A). Compared to *SIRT6^fl/fl^* mice, the cKO mice exhibited impaired mucosal healing at the TES (Figure 3B). Micro‐CT results also indicated a significant decrease in trabecular bone quantity and bone density within the TES of the cKO mice, accompanied by a reduction in BMD (Figure 3C,D). Histological staining revealed that the cKO mice exhibited an enlargement of necrotic areas compared to the control group (Figure 3E), along with a significant increase in infiltration of inflammatory cells such as M1 macrophages and neutrophils (Figure 3G). The analyses of lymphatic vessel morphology and function revealed a significant reduction in the quantity of Lyve1^+^ cells, as well as an impaired ability to form a continuous tubular lymphatic drainage system in the cKO mice (Figure 3F). Functional experiments also confirmed a significant reduction in ICG clearance rates within the buccal periodontal tissues of the cKO mice (Figure 3I). Additionally, the drainage of Dil‐labeled M1 macrophages was also observed to decrease within the submandibular lymph nodes of the cKO mice, indicating impaired lymphatic drainage function (Figure 3H). In summary, the conditional knockout of *SIRT6* impaired lymphatic drainage function by promoting ERS‐induced apoptosis in LECs, thereby exacerbating BRONJ.

### 2.4 Rapamycin alleviates ERS‐induced apoptosis through activating autophagy activation

**FIGURE 4:**
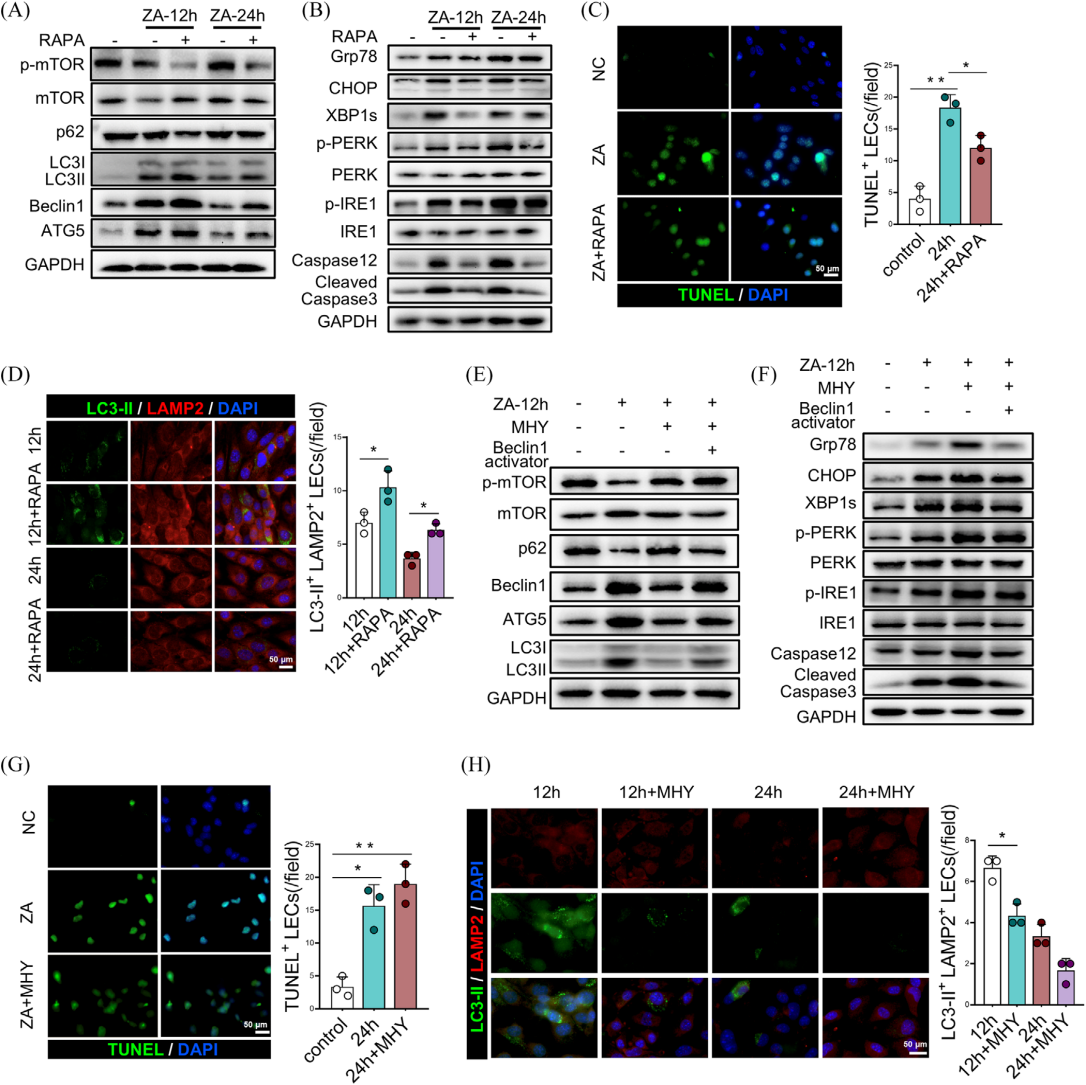
Rapamycin inhibits the mTOR pathway to alleviate endoplasmic reticulum stress (ERS)‐induced apoptosis in lymphatic endothelial cells (LECs). Western blot analysis of the protein expression of autophagy‐related pathways (A) and ERS pathways (B) in LECs after 10  nM RAPA treatment. (C) Immunofluorescence staining to observe the effect of RAPA on apoptotic LECs proportion induced by Zoledronate acid (ZA). (D) Immunofluorescence staining to observe the effect of RAPA on the number of autolysosomes in LECs. Western blot analysis of autophagy‐related pathways (E) and ERS pathways (F) in LECs after treatment with mTOR activator MHY and Beclin1 agonist. (G) Immunofluorescence staining to observe the effect of MHY on the proportion of apoptotic LECs. (H) Immunofluorescence staining to observe the effect of rapamycin on the number of autolysosomes in LECs treated with mTOR activator MHY and Beclin1 agonist. A total of three subjects were analyzed. The results are presented as the mean ± standard deviation. *p < .05; **p < .01.

Considering that ERS can initiate autophagy to remove damaged proteins and organelles, thereby alleviating further exacerbation of ERS and maintaining cellular homeostasis, we aimed to prevent ERS‐induced apoptosis in ZA‐treated LECs by using the autophagy activator RAPA. Following a 12 and 24‐h treatment with 10  nM RAPA, the combination of RAPA and ZA group demonstrated significantly enhanced expression of autophagy protein ATG5 and anti‐apoptotic protein Beclin1 compared to the ZA alone and untreated groups. Additionally, the expression of autophagy inhibitory protein p‐mTOR/mTOR and autophagy adaptor protein p62 decreased (Figure 4A and Figure S4A). Similarly, a significant downregulation in the expression of GRP78, XBP1s and p‐PERK/PERK was found in the combination of the RAPA and ZA groups. Apoptosis‐related indicators, CHOP, Caspase‐12 and Cleaved Caspase3 also exhibited similar trends (Figure 4B and Figure S4B). Furthermore, the immunofluorescence (IF) results further confirmed that RAPA effectively attenuated the apoptotic rate of LECs induced by ZA (Figure 4C), while simultaneously increasing the formation of autolysosome (Figure 4D). Besides, we co‐administered the mTOR activator MHY and the Beclin1 agonist BFL to modulate the autophagy pathway. The results showed that the combination of MHY and ZA group effectively activated p‐mTOR while suppressing ATG5 and Beclin1 expression compared to the ZA group (Figure 4E and Figure S4C). Furthermore, it upregulated the ERS response and apoptotic protein Caspase12 in LECs (Figure 4F and Figure S4D). IF staining further confirmed that MHY increased the proportion of apoptotic LECs induced by ZA and inhibited the formation of autolysosome (Figure 4G,H). Conversely, the co‐administration of BFL and ZA promoted the expression of ATG5 and Beclin1 and inhibited LECs apoptosis (Figure 4E,F and Figure S4C,D). Together, the activation of the autophagy pathway can effectively relieve the ERS‐apoptosis effect in LECs and maintain cellular homeostasis.

### 2.5 *Lyve1^creERT^*; *ATG5^fl/fl^* mice exhibit impaired lymphatic drainage and aggravated BRONJ

**FIGURE 5:**
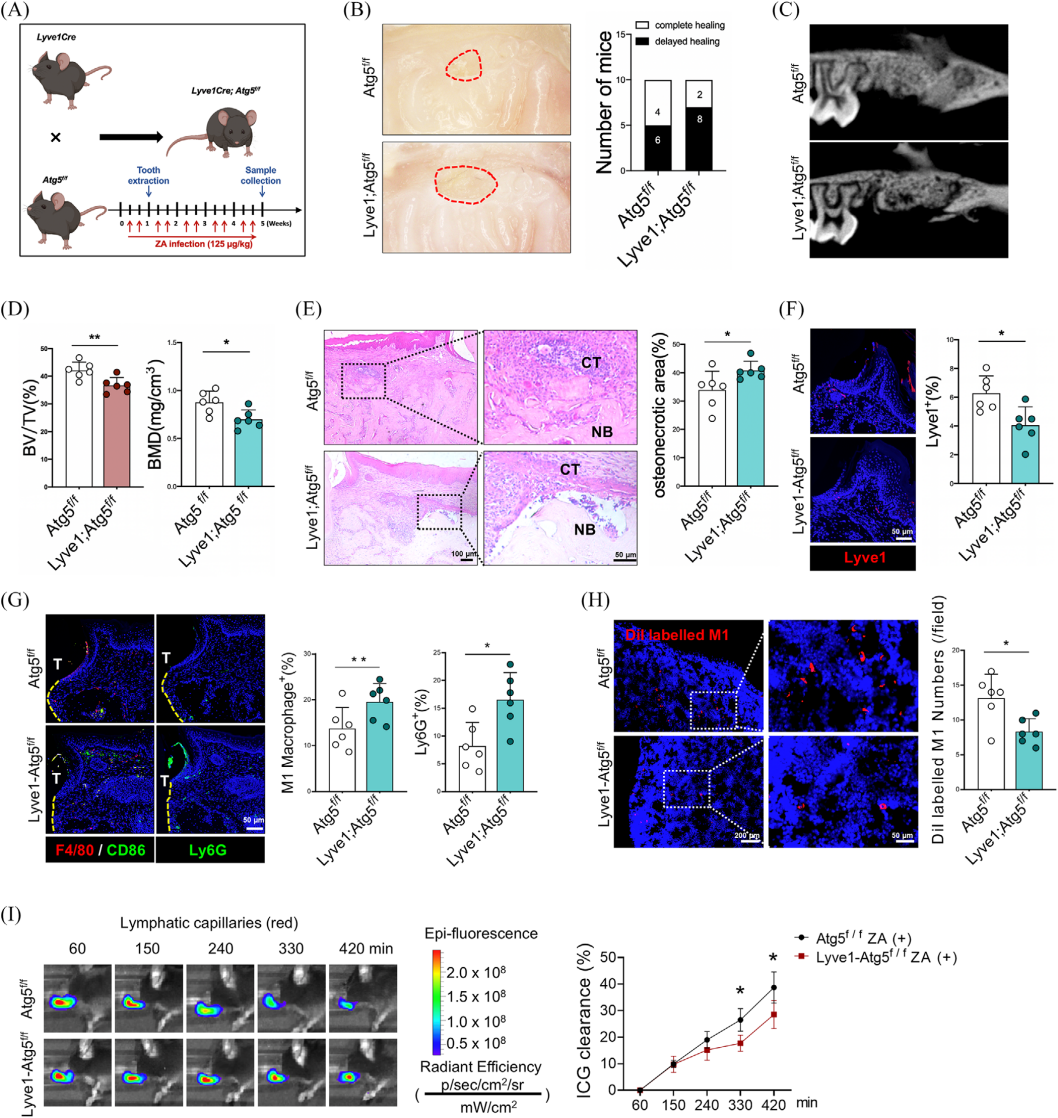
*Lyve1^creERT^; ATG5^fl/fl^* mice exhibit exacerbated BRONJ phenotype and lymphatic reflux dysfunction. (A) Schematic representation of the construction of *Lyve1^creERT^; ATG5^fl/fl^* mice. (B) The gross morphology of the tooth extraction sockets was observed. (C) Micro‐CT imaging analysis of tooth extraction sockets healing. CT, connective tissue; NB, necrotic bone. (D) Micro‐CT measurements of bone volume (BV) / tissue volume (TV) and bone mineral density (BMD). (E)HE staining to observe histological morphology and osteonecrosis of tooth extraction sockets. (F) Immunofluorescence staining to observe lymphatic vessels labelled with Lyve1. (G) Immunofluorescence staining to observe the infiltration of neutrophils and macrophages in mucosal tissue of tooth extraction sites. (H) Immunofluorescence staining to observe macrophages labelled with Dil in submandibular lymph nodes. (I) Lymphatic reflux functional experiments in the TES. A total of six subjects were analyzed. The results are presented as the mean ± standard deviation. *p < .05; **p < .01.

Given the crucial role of the autophagy pathway in apoptotic stress, we generated *Lyve1^creERT^; ATG5^fl/fl^* mice (cKO) to establish a BRONJ model by specifically deleting *ATG5* in LECs to inhibit the autophagy pathway (Figure 5A). Compared to *ATG5^fl/fl^* mice, delayed mucosal healing was observed following tooth extraction in the cKO mice (Figure 5B). The cKO mice also exhibited a decreased trabecular bone density and quantity within the TES (Figure 5C,D). HE) staining showed an enlarged area of necrotic bone in the cKO mice (Figure 5E). Besides, assessment of the inflammatory levels within the TES revealed a significant increase in the infiltration of inflammatory cells such as M1 macrophages and neutrophils (Figure 5G).

Subsequently, we analyzed the impact of conditional *ATG5* knockout on lymphatic vessel morphology and function. The IF results indicated a significant reduction in Lyve1^+^ cells within the TES in the cKO mice, resulting in impaired formation of continuous lymphatic vessels (Figure 5F). The analyses of lymphatic drainage function also demonstrated a significant reduction in ICG clearance rate (Figure 5I). Similarly, the migration of Dil‐labeled M1 macrophages to the submandibular lymph nodes was delayed in the cKO mice, indicating impaired lymphatic drainage function (Figure 5H). Collectively, the conditional knockout of *ATG5* in LECs exacerbated BRONJ by modulating lymphatic function, leading to a suppression of autophagy.

### 2.6 ZDPR alleviates ERS‐induced apoptosis by inhibiting the mTOR pathway

**FIGURE 6:**
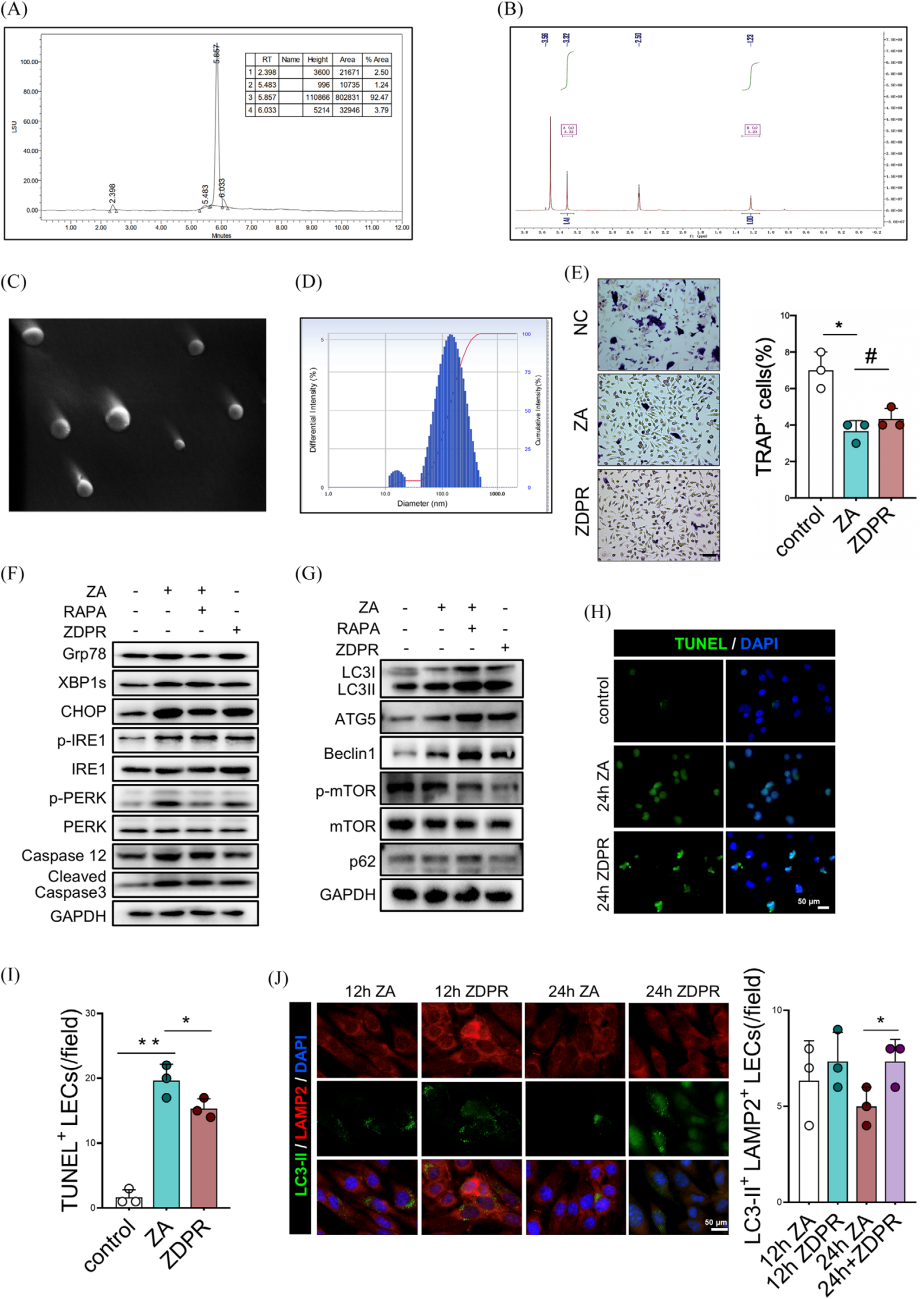
ZDPR loaded with RAPA inhibits mTOR pathway in vitro to alleviate endoplasmic reticulum stress (ERS)‐ induced apoptosis in lymphatic endothelial cells (LECs). (A) High‐performance liquid chromatography (HPLC) analysis of ZDPR. NMR spectroscopy (B) and SEM (C) used to characterize the chemical structure and surface morphology of the ZDPR. (D) Dynamic light scattering to measure the particle diameter of ZDPR. (E) TRAP staining of osteoclast induction for 7 days with Zoledronate acid (ZA) or ZDPR. Western blot analysis of ERS pathways (F) and autophagy pathways (G) in LECs after ZA or ZDPR treatment. (H) Immunofluorescence staining to detect the proportions of apoptotic LECs and quantitative analysis (I). (J) Immunofluorescence staining to detect the quantities of autolysosomes in LECs (J). A total of three subjects were analyzed. The results are presented as the mean ± standard deviation. # not statistically significant, *p < .05; **p < .01.

Given that RAPA significantly mitigated lymphatic dysfunction and osteonecrosis, we attempted to devise a bone‐targeted autophagy activator to alleviate BRONJ. The synthetic route is depicted in the accompanying diagram (Figure S5A). We selected DSPE‐PEG‐NH_2_ as the drug delivery vehicle and conjugated the reactive amino group of DSPE‐PEG‐NH_2_ with ZA to form ZA‐DSPE‐PEG conjugates, whose chemical structure was validated via NMR spectroscopy. The ^1^H NMR spectrum of DSPE‐PEG‐ZA displayed characteristic peaks that confirmed its structure: a prominent peak at 3.56 ppm corresponding to the ethylene glycol (PEG) chain, a peak at 1.23 ppm attributed to the terminal methyl groups of the DSPE fatty acid chains, and minor signals at 2.50 ppm from the glycerol backbone, with a residual solvent peak at 3.32 ppm. These peaks collectively confirm the presence of PEG, DSPE and ZA components in the final conjugate (Figure 6B). To further ensure the purity and chemical composition of DSPE‐PEG‐ZA, we performed high‐performance liquid chromatography (HPLC) and Fourier‐transform infrared (FT‐IR) spectroscopy. The HPLC results revealed a single prominent peak constituting 92.47% of the total peak area, indicating high purity (Figure 6A). FT‐IR analysis confirmed the presence of key functional groups, with characteristic absorption bands observed at 1750 cm⁻¹ (C = O stretching of ester groups), 2930 cm⁻¹ (C‐H stretching in DSPE fatty acid chains), 1100 cm⁻¹ (C‐O‐C stretching of the PEG backbone) and 950 cm⁻¹ (P‐OH stretching in phosphonic acid groups). These results confirm the successful conjugation of DSPE‐PEG with ZA (Figure S5B).

Subsequently, RAPA was loaded into the tetrahydrofuran (THF) solution to generate ZA‐DSPE‐PEG‐RAPA nanoparticles (hereafter referred to as ZDPR). To characterize the fundamental properties of the drug, scanning electron microscopy (SEM) and dynamic light scattering (DLS) were employed to observe the surface morphology and particle diameter of ZDPR. ZDPR exhibited uniform nanoscale particle morphology (Figure 6C), with an average particle diameter of approximately 111.9  nm (Figure 6D). For dose‐response studies, we utilized LECs in vitro to evaluate ZDPR concentration effects, observing reduced cell viability at higher concentrations via Cell Counting Kit‐8 assays, consistent with ZA's cytotoxicity profile (Figure S5C). The analysis of ERS markers showed dose‐dependent increases in GRP78 and pIRE1/IRE1, while GRP78, pIRE1/IRE1, XBP1s and Cleaved Caspase‐3 activation were less pronounced than with ZA, suggesting that ZDPR induces ERS in a dose‐dependent but less toxic manner (Figure S5D). Primary bone marrow‐derived macrophages were extracted in vitro and induced with macrophage colony‐stimulating factor (M‐CSF) and receptor activator of nuclear factor‐κB ligand (RANKL) for osteoclast differentiation. TRAP staining results indicated that the inhibitory effect of bisphosphonates on osteoclast differentiation was preserved by ZDPR (Figure 6E). Furthermore, we assessed the impact of ZDPR on autophagy and ERS pathways and found that treatment with ZDPR alone had similar effects to the combined use of ZA and RAPA. It attenuated the ERS response and Caspase‐12 expression induced by ZA (Figure 6F), while concurrently promoting the autophagy pathway and Beclin1 expression (Figure 6G). Tunnel staining further confirmed that ZDPR treatment reduced the ratio of apoptotic LECs induced by ZA (Figure 6H,I) while increasing autolysosome production (Figure 6J).

### 2.7 ZDPR promotes lymphatic drainage to alleviate inflammation and osteonecrosis

**FIGURE 7:**
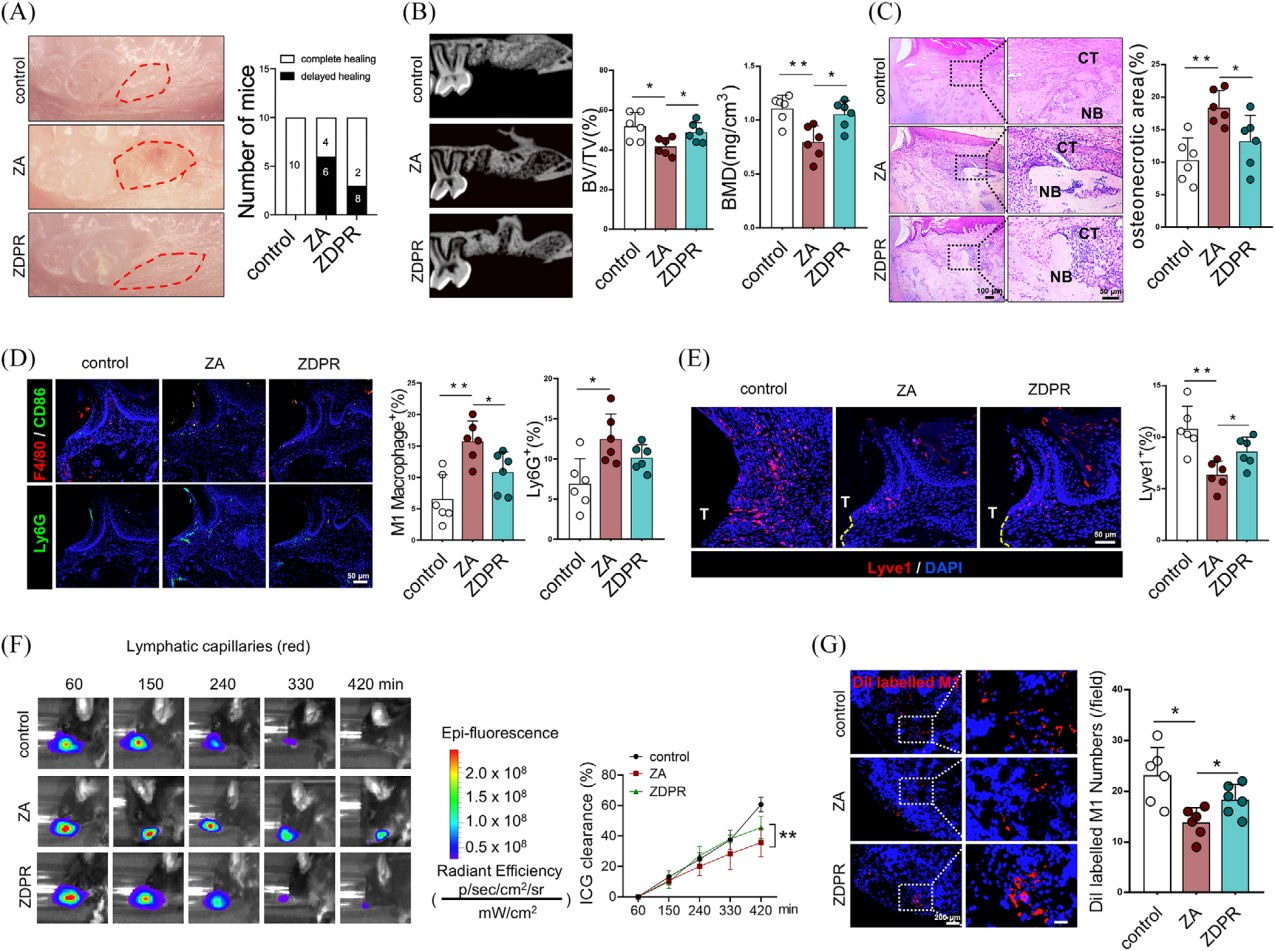
ZDPR promotes lymphatic drainage in vivo, alleviating local inflammation and relieving BRONJ. (A) The gross morphology of the tooth extraction sockets in mice treated with Zoledronate acid (ZA) or ZDPR was observed. (B) Micro‐CT analysis of tooth extraction sockets. (C) Histological morphology of tooth extraction sockets observed through HE staining. CT, connective tissue; NB, necrotic bone. (D) Immunofluorescence staining to observe the infiltration of neutrophils and macrophages. (E) Immunofluorescence staining to observe lymphatic vessels labelled with Lyve1. T, tooth. (F) The functional experiments of lymphatic vessels in the TES. (G) Dil‐labeled macrophages were observed by Immunofluorescence staining. A total of six subjects were analyzed. The results are presented as the mean ± standard deviation. *p < .05; **p < .01.

We administered ZDPR via tail vein injection to assess its impact on the healing of TES at the same concentration as ZA. The ZDPR group showed normal mucosal healing in the TES compared to mice treated with ZA (Figure 7A). Micro‐CT also revealed that the ZDPR group exhibited increased formation of new bone trabeculae within the extraction sockets compared to the ZA‐treated group, with the BV/TV more closely resembling that of the control group (Figure 7B). Moreover, HE staining demonstrated that the ZDPR group had fewer necrotic bones than the ZA group (Figure 7C). Meanwhile, there was a notable decrease in pro‐inflammatory macrophages and neutrophils in the gingival mucosa of the ZDPR group (Figure 7D), while the number of Lyve1^+^ cells increased (Figure 7E). The functional experiment also confirmed that ZDPR treatment can avoid the local lymphatic reflux obstruction in the jawbone (Figure 7F). Additionally, the drainage rate of Dil‐labeled M1 macrophages was significantly elevated in the ZDPR group (Figure 7G). Furthermore, we evaluated the effects of ZDPR in *Lyve1^creERT^*; *SIRT6^fl/f^* and *Lyve1^creERT^*; *ATG5^fl/f^* mice. HE staining showed that ZDPR significantly enhanced tooth socket healing in both conditional knockout models, with reduced necrotic bone and decreased inflammatory cell infiltration in the extraction sites (Figure S6A,B). HE staining of principal organs, including the heart, liver, spleen, lung and kidney, indicated no significant off‐target effects, confirming both its safety and therapeutic potential (Figure S6C). These results reveal that our novel drug ZDPR effectively mitigates the potential adverse effects of bisphosphonates on jaw bone necrosis, while simultaneously inhibiting osteoclast differentiation.

## 3 DISCUSSIONS

**FIGURE 8:**
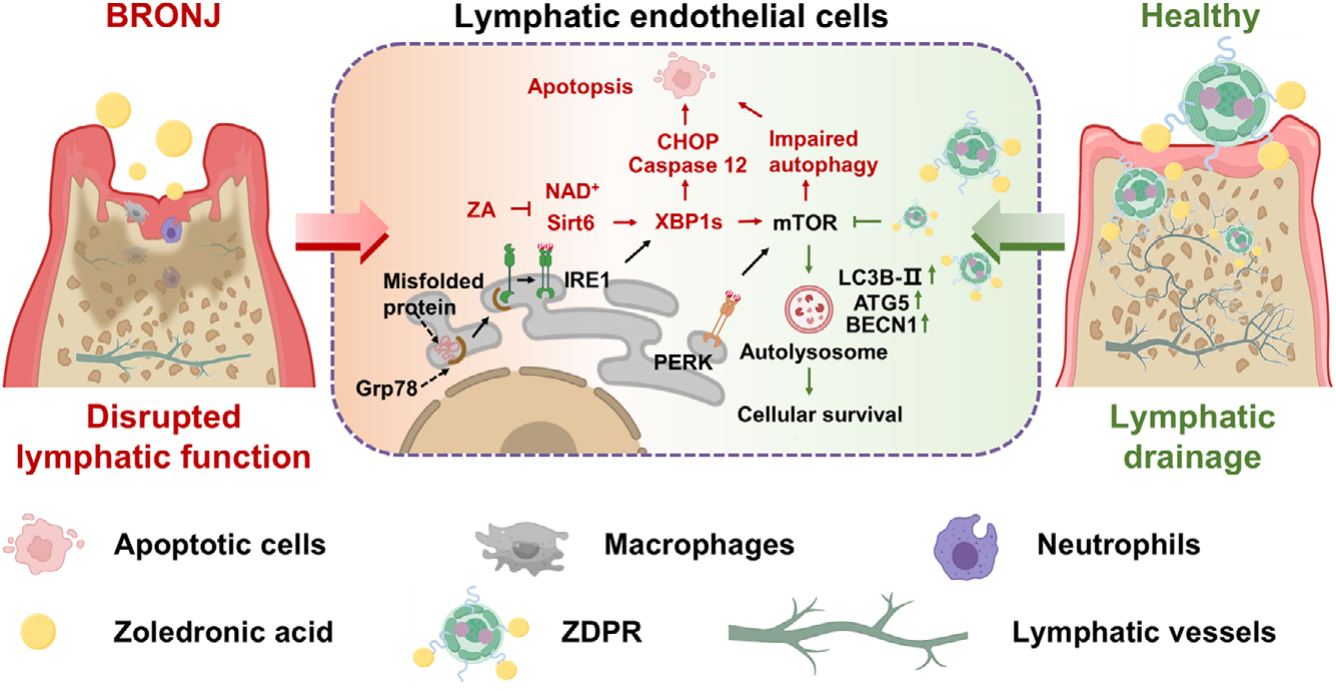
**Schematic diagram**: We propose the following working model based on our results. Continuous Zoledronate acid (ZA) stimulation inhibits the NAD^+^/SIRT6 pathway in lymphatic endothelial cells (LECs), promoting XBP1s activation and subsequent endoplasmic reticulum stress (ERS)‐induced apoptosis. This process impairs lymphatic vessel integrity and drainage function, exacerbating inflammation accumulation and contributing to the progression of bisphosphonates‐related osteonecrosis of the jaw (BRONJ). Innovative nanoparticle ZDPR alleviates ERS by activating autophagy, reducing the proportion of apoptotic LECs, promoting lymphatic reflux, and ameliorating BRONJ symptoms.

Our study has revealed significant insights into the inherent relation among LECs apoptosis, impaired lymphatic drainage and ZA‐induced BRONJ, highlighting the pivotal role of ZA in promoting ERS‐induced apoptosis in LECs. The main contributions consisted of the following three aspects. First, by employing tissue clearing techniques and RNA‐seq analysis, we have demonstrated that ERS‐induced apoptosis in ZA‐treated LECs detrimentally impacts the functionality of lymphatic drainage, consequently resulting in the accumulation of inflammation in the jawbone. Secondly, using two conditional knockout mouse models and ZA‐treated LECs, we elucidated the molecular mechanism through which ZA regulated the balance between ERS and autophagy via the NAD^+^/ Sirt6/XBP1s axis. Thirdly, we developed a novel drug, ZDPR, which effectively alleviated ERS‐induced apoptosis through the inhibition of the mTOR pathway, facilitated lymphatic drainage and thereby alleviated BRONJ. The findings have provided a better understanding of the mechanisms of BRONJ and potential avenues for its prevention and treatment (Figure 8).

Lymphatic vessels and blood vessels intertwine to form an extensive transport network within the bone microenvironment.^30^ While it is well‐established that intra‐bony blood vessels regulate osteogenesis and hematopoiesis,^31^ the impact of lymphatic vessels on bone repair remains uncertain. Biswas et al. recently identified a network of lymphatic vessels within the bone marrow cavity and discovered that during lymphangiogenesis, proliferating lymphatic endothelial cells secrete CXCL12, which is crucial for hematopoietic and bone regeneration.^13^ This innovative work reveals the role of lymphatic secretion in mitigating bone damage. Our research illustrated the pivotal role of lymphatic vessels in facilitating immune cell trafficking during BRONJ. We provided evidence that apoptosis induced by ZA in LECs significantly compromised the structural integrity and lymphatic drainage, leading to subsequent accumulation of inflammation and progression of BRONJ. Similarly, enhanced lymphatic drainage promotes tibial fracture healing and musculoskeletal system repair.^32^, ^33^ Our study was the first to highlight the crucial role of the lymphatic system in jawbone repair and the development of BRONJ.

ZA, a potent bisphosphonate, plays a crucial role in inhibiting bone resorption. It acts by binding to hydroxyapatite in bone, thereby inhibiting osteoclast‐mediated bone resorption. ZA also induces osteoclast apoptosis and disrupts the mevalonate pathway, which is essential for osteoclast function, thus contributing to its anti‐resorptive effects.^34^, ^35^, ^36^ Recent findings have shown the participation of ER stress in ZA‐induced BRONJ and that ZA reduces cell viability in a concentration‐dependent manner and elevates both the oxidative stress index and ER stress, indicating significant cellular stress.^18^, ^37^, ^38^ ER stress is a critical cellular process maintained through the unfolded protein response. When the ER's protein folding capacity is insufficient to cope, UPR is activated to restore normal function by enhancing protein folding capacity, degrading misfolded proteins and reducing protein translation.^39^, ^40^ However, under persistent or severe ER stress, apoptosis can be initiated via both intrinsic and extrinsic pathways.^41^ The most important findings of our study are that ERS is strongly involved in ZA‐induced LECs apoptosis and BRONJ. Namely, we showed that ZA‐induced persistent ERS in LECs, triggered apoptosis and affected lymphatic function.

Upon detecting ER stress, PERK oligomerizes, phosphorylating itself and the ubiquitous translation initiation factor eIF2α, which leads to the indirect inactivation of eIF2α and the inhibition of mRNA translation. Through this process, PERK reduces the influx of protein into the ER to relieve stress.^42^, ^43^ XBP1s, another key player in UPR, also plays a crucial role in ER stress‐induced apoptosis. When spliced and activated, XBP1s upregulate genes that assist in protein folding and degradation, but if stress persists, it can contribute to apoptotic signalling pathways. Furthermore, XBP1s is a key transcription factor in the UPR regulated by Sirt6,^26^ which controls genes related to protein folding, secretion and degradation, playing a crucial role in maintaining ER homeostasis.^44^ Our study showed that Sirt6 enhanced UPR and activated autophagy by inhibiting XBP1s, thereby reducing apoptosis in LECs. Sirt6's dual regulatory role in ERS and autophagy highlights its therapeutic potential in persistent ERS and chronic inflammatory diseases.

ZA has been shown to increase the levels of autophagy‐related proteins such as LC3B‐II and autophagosomes in breast cancer cells and colon cancer cells.^45^, ^46^ This regulation of autophagy was also linked to the promotion of apoptosis and the inhibition of cell proliferation, highlighting the dual role of ZA in cancer cell management. Additionally, ZA‐induced autophagy was found to be both dose‐ and time‐dependent, and this process was associated with increased oxidative stress.^47^, ^48^ Here, we discovered the transition of autophagy from early activation to late inhibition is the key to LECs apoptosis. The inhibition of mTOR triggers ULK1 to initiate the formation of autophagosomes, with *ATG5* playing a pivotal role in this process.^49^ Using *ATG5* conditional knockout models, we demonstrated that autophagy inhibition exacerbated BRONJ by further impairing lymphatic function. Previous research has demonstrated that autophagy can prevent apoptosis, thereby promoting cell survival.^50^ When autophagy is inhibited, it can interact with ERS to induce apoptosis.^51^ Our study also confirmed that ZA triggered autophagy in the early period of ERS, while constant stimulation of ZA shifted LECs from autophagy to apoptosis, and increased autophagy induced by RAPA could reverse ERS‐induced apoptosis in LECs. Ketamine, often used as an anaesthetic, has been shown to influence ER stress and autophagy in a dose‐dependent and time‐dependent manner,^52^, ^53^ primarily at high doses or with prolonged exposure.^54^, ^55^ Given the brief, low‐dose application of ketamine in our tooth extraction procedure, we consider its impact minimal compared to the sustained effects of ZA in inducing BRONJ pathology. Nonetheless, additional studies could further clarify ketamine's potential interactions with ER stress and autophagy pathways in lymphatic endothelial cells.

We emphasized that autophagy was vital for LECs' survival under ERS conditions, and its inhibition may worsen ERS‐induced apoptosis and contribute to lymphatic drainage insufficiency. The clinically well‐established drug RAPA significantly induces autophagy, thereby promoting the initiation of this cellular process and enhancing stress response mechanisms. It is widely utilized as an immunosuppressant to prevent organ transplant rejection and as an anti‐proliferative agent in certain cancer therapies, demonstrating its efficacy and versatility in modulating cellular processes. Consequently, we developed nanoparticle‐based ZA and RAPA (ZDPR) as a potential preventive measure for BRONJ. ZDPR not only preserved anti‐osteoclastic activity but also reduced LECs apoptosis, promoting lymphatic drainage and alleviating the inflammatory microenvironment in the jawbone. The dual action of ZDPR highlighted its therapeutic promise in preventing and treating bisphosphonate‐related complications.

Recognizing the potential toxicity associated with RAPA, we explored additional therapeutic approaches to alleviate ER stress. Chemical chaperones such as 4‐phenylbutyric acid and tauroursodeoxycholic acid facilitate correct protein folding and have been shown to reduce ER stress‐induced damage in various disease models,^56^, ^57^ including metabolic disorders and inflammatory conditions.^58^, ^59^ Furthermore, SIRT activators like resveratrol mitigate ER stress by activating the deacetylase SIRT1, which is demonstrated to play a key role in limiting ER stress.^60^ Our data indicate that NAD^+^ supplementation may alleviate ZA‐induced ER stress and apoptosis. In fact, administering the SIRT6 agonist MDL‐800 significantly improved bone healing and reduced inflammation in ZA‐induced BRONJ models. Based on findings from previous studies, possible mechanisms include enhancing SIRT6 activity to promote autophagy and protein folding, thereby reducing cellular stress^27^; upregulating autophagy‐related proteins (such as LC3 and Beclin1) to facilitate the clearance of misfolded proteins and damaged organelles^61^; and supporting mitochondrial function, which can lower oxidative stress and decrease mitochondrial‐mediated apoptosis.^62^ Additionally, protein degradation pathway activators, such as the proteasome inhibitor MG132, promote ER‐associated degradation and autophagy, thereby alleviating ER stress and reducing apoptosis.^63^ These alternative modulators broaden the scope of potential strategies for targeting ER stress‐related pathologies in BRONJ.

In conclusion, our study has provided critical insights into the mechanisms underlying ZA‐induced ERS and apoptosis in LECs, which disrupts lymphatic function and promotes the progression of BRONJ. Based on the targeted autophagy activity of ZDPR, this offered a promising approach to counteract the adverse effects of bisphosphonates. Nonetheless, further research is necessary to fully elucidate the mechanisms by which ZDPR mitigates ER stress and promotes lymphatic drainage.

## 4 MATERIALS AND METHODS

### 4.1 Animal model

According to the guidelines of the Animal Care Committee of the School of Stomatology, Nanjing Medical University, all experiments were approved by the Ethics Committee (Approval No. IACUC‐1805006 and IACUC‐2308037). *Lyve1^creERT^* (Cat#T007654) and *ATG5f/f *(Cat#T006660) mice were sourced from Jiangsu Jicui Yaokang Biotechnology Co., Ltd., and *SIRT6f/f *(JAX# 017334) mice from Jackson Laboratory. We obtained 8‐week‐old C57BL/6N mice from Nanjing Medical University for our studies. *ATG5^f/f ^* and *Lyve1^creERT^* mice were crossed to generate *Lyve1^creERT^* mice with *ATG5^f/+^* heterozygotes. Further crossing of *Lyve1^creERT^*; *ATG5^f/+^* with *ATG5^f/f^* mice resulted in homozygous conditional *ATG5* knockout mice (*Lyve1^creERT^*; *ATG5^f/f^*). *Lyve1^creERT^*; *SIRT6^f/f^* mice were generated using a similar process. Animals were kept at 22°C and continually exposed to light and dark allowing for ad libitum availability of food and water.

The BRONJ‐like model in mice was established as previously detailed.^64^ Each group of mice received 125 µg/kg ZA (Novartis Oncology) or phosphate‐buffered saline (PBS) via tail veins twice a week for five consecutive weeks (*n* = 6). The 125 µg/kg dose, approximately double the oncologic dose of 66 µg/kg, was chosen to increase the incidence of BRONJ, reflecting the dose‐dependent effect observed in humans. Ten ZA doses were administered over 5 weeks. SIRT6 activator MDL‐800 (150 mg/kg) was administered intraperitoneally to the mice every two days throughout the experimental period. An intraperitoneal ketamine (100 mg/kg) injection was given 1 week after the initial ZA injection for the extraction of the first right maxillary molar.

### 4.2 Cell culture

The mouse lymphatic endothelial cells SVEC4‐10 (CRL‐2181) were cultured in Dulbecco's Modified Eagle Medium (DMEM) with 10% fetal bovine serum (FBS) and antibiotics from the American Type Culture Collection. As described in our previous study,^65^ bone marrow cells were flushed from femurs and tibias of C57BL/6 mice and cultured for 4 h in DMEM with 2% FBS and antibiotics. After the collection of non‐adherent cells, complete DMEM with 10% FBS, antibiotics, and murine M‐CSF (10 ng/mL) was used to culture the cells for 3 days. Osteoclast differentiation was accomplished by culture of bone marrow cells in complete DMEM containing 20 ng/mL RANKL. 100 µM OSS_128167 (MedChemExpress, HY‐107454) was used as a Sirt6 inhibitor. 1 µM MAC8866 (MedChemExpress, HY‐104040) was used as a XBP1s inhibitor. MHY1485 (MedChemExpress, HY‐B0795) was used to inhibit autophagy at the concentration of 1 µM. 10 µM Tat‐beclin 1 (MedChemExpress, HY‐P2260) was used as a Beclin1 agonist.

### 4.3 RNA‐seq analysis

The RNA quality for sequencing was evaluated through agarose gel electrophoresis and Nanodrop measurements. Subsequently, an RNA library was prepared with the U‐mRNAseq Library Pre Kit, and profiling was conducted on the Illumina Novaseq 6000 platform utilizing sequencing by synthesis methodology. Quantitative RNA‐seq data analysis was performed using HTSeq software, and edgeR software was utilized to ascertain differential gene expression.

### 4.4 LC‐tandem mass spectrometry data analysis

Samples were transferred into EP tubes and mixed with an extraction solution containing isotopically labelled internal standards. After vortexing for 30 s, the mixture was sonicated, followed by incubation at ‐40°C for 1 h. Samples were then centrifuged for 15 min, and the supernatant was collected into vials for subsequent analysis. Target compounds were chromatographically separated with a Vanquish ultra‐HPLC system (Thermo Fisher Scientific) that is equipped with a Waters ACQUITY UPLC BEH Amide column. Mass spectrometric analysis was performed on an Orbitrap Exploris 120 mass spectrometer controlled by Xcalibur software (version 4.4; Thermo), enabling data collection for both primary and secondary mass spectrometry.

### 4.5 Micro‐CT

The maxilla of mice was scanned using high‐resolution micro‐CT at 55 kV energy and 456 µA current, achieving a voxel size of 15.6 µm. Subsequent three‐dimensional reconstruction of the maxilla was performed using NRecon v1.6 and analyzed with CTAn v1.13.8.1. The extraction socket of the first molar (TES) was designated as the region of interest for analysis. The healing of TES was evaluated using parameters BV/TV and BMD.

### 4.6 Flow cytometry analysis

We harvested cells in TES, segmented them into small portions and digested them for 30 min with collagenase type I (Sigma) at 37°C. Cells were collected in PBS/1% bovine serum albumin and passed through a 40 µm strainer. RBCs were lysed using RBC lysis buffer. Single‐cell suspensions were sequentially stained with the corresponding antibodies given in Table S2. Results were analyzed using Flow Jo Software (Version 10.8.1; FLOWJO, LLC).

### 4.7 PEAGASOS tissue clearing method

Mice were anaesthetized and perfused intracardially with 0.02% heparin in PBS, followed by 4% paraformaldehyde. The samples underwent decalcification in a 0.5 M ethylenediaminetetraacetic acid (EDTA) solution (pH 8.0) for 4 days and then bleached in 25% Quadrol (v/v in water) at 37°C for 1 day. Lipid removal was performed at 37°C using 30%, 50% and 70% *tert*‐butanol (tB) solutions with constant shaking. Subsequently, dehydration was achieved using a tB‐PEG solution (75% tB, 22% PEG MMA500 and 3% Quadrol) at 37°C, followed by clearing in BB‐PEG medium (75% BB, 22% PEG‐MMA500 and 3% Quadrol) until the samples became transparent. The samples were stored at room temperature in a BB‐PEG medium. Images were acquired using light sheets of different wavelengths (488 and 561 nm) and analyzed with Imaris software (version 9.0.1; Bitplane).

### 4.8 HE and IF staining

To perform routine HE and IF staining on mouse maxillae, 4% paraformaldehyde was applied, followed by decalcification with EDTA and then sectioning into 6 µm thick sections. Sections were subjected to deparaffinization in xylene before being rehydrated in alcohol, rinsed in PBS and stained with HE. In order to stain sections with antibodies against LYVE‐1, F4/80, CD86 and Ly6G, sections were incubated with goat serum to block non‐specific binding, followed by staining with the antibodies at 4°C overnight. After incubation with Cy3 or fluorescein isothiocyanate‐labeled secondary antibodies for 1 h, the sections were stained with DAPI to visualize nuclear structures. Observations were made using a *Leica* fluorescence microscope (*Leica*).

### 4.9 Transmission electron microscopy

LECs were cultured overnight and treated with ZA for different times (0, 12, 18 and 24 h). Cells were then digested with 0.25% trypsin, centrifuged, rinsed with PBS and fixed with 1% glutaraldehyde for 30 min in the dark. Samples were processed for transmission electron microscopy analysis, including fixation with osmium acid, dehydration, permeation, embedding, ultrathin sectioning and staining with lead acetate. A JEM‐1400Flash transmission electron microscope (JEOL Corporation) was used for imaging.

### 4.10 TUNEL staining

TUNEL staining was performed to assess LEC apoptosis according to the manufacturer's protocol (abs50047; *Absin*). Cells were fixed with 4% paraformaldehyde for 30 min, and incubated with TdT enzyme solution for 1 h at 37°C. TUNEL‐positive cells were imaged using a *Leica* inverted fluorescence microscope. Semi‐quantitative analysis was performed on at least 5 slices per sample and three random fields per slice.

### 4.11 Synthesis of ZDPR

To synthesize the ZA‐DSPE‐PEG‐RAPA conjugate, 100 mg of ZA was dissolved in distilled dimethylformamide with triethylamine (TEA) and transferred to a sealed container under a nitrogen atmosphere. Subsequently, 90 mg of carbonyldiimidazole (CDI) was added to the solution, and the reaction mixture was stirred continuously at 60°C in an oil bath for 24 h. After the reaction, TEA was distilled out using a rotary evaporator, causing the activated ZA to precipitate. The precipitate was collected by centrifugation, washed twice with acetonitrile to remove any residual CDI and then dried in a rotary evaporator to obtain purified activated ZA. In the next step, 1 g  of DSPE‐PEG‐NH_2_ and 22.6 mg of activated ZA were dissolved in DMSO with TEA under a nitrogen atmosphere and allowed to react for 12 h in a sealed container. The resulting DSPE‐PEG‐ZA conjugate was purified using column chromatography with acetonitrile as the mobile phase and dried using a rotary evaporator. Finally, the DSPE‐PEG‐ZA conjugate was dissolved in water, and a THF solution of RAPA was added dropwise. The THF was replaced by nitrogen gas to yield the final product, ZA‐DSPE‐PEG‐RAPA. This method ensured the successful conjugation of ZA to DSPE‐PEG and the subsequent loading of RAPA, resulting in the nanoparticles used in our experiments. Field‐emission scanning electron microscopy (SEM, Auriga; Zeiss) was used to evaluate the morphology and mesoporous structure.

### 4.12 Nuclear magnetic resonance analysis

Nuclear magnetic resonance (NMR) spectroscopy was performed to confirm the chemical structure and composition of the synthesized DSPE‐PEG‐ZA conjugate. ^1^H NMR spectra were obtained using a Bruker AVANCE 400 MHz NMR spectrometer. Samples were dissolved in deuterated dimethyl sulfoxide (DMSO‐d6) to achieve a final concentration of approximately 10 mg/mL. The ^1^H NMR spectra were recorded at 25°C with a standard 5 mm NMR tube. The NMR spectra were processed and analyzed using Bruker's TopSpin software, which allowed for detailed peak assignment and confirmation of the final conjugate structure.

### 4.13 HPLC analysis

HPLC was performed using a reverse‐phase C18 column (4.6 × 150 mm, 5 µm). The mobile phase included water with 0.1% TFA (A) and acetonitrile with 0.1% TFA (B), utilizing a gradient: 0–5 min at 30% B, increasing to 95% B by 20 min, maintaining 95% B until 25 min, then returning to 30% B by 30 min, with 5 min for re‐equilibration. Detection was achieved with a Waters 2424 ELSD (evaporation temperature 40°C, carrier gas flow 2.0–3.0 L/min) for PEG and lipid components.

### 4.14 FT‐IR analysis

The chemical structure of DSPE‐PEG‐ZA was characterized using FT‐IR with an IRPrestige‐21 (Shimadzu). Approximately 1–2 mg of the sample was mixed with potassium bromide (KBr) powder at a ratio of 1:100 and pressed into a thin pellet. Spectra were recorded over the range of 4000–400 cm⁻¹ with a resolution of 4 cm⁻¹, and each spectrum was averaged over 32 scans to enhance the signal‐to‐noise ratio.

### 4.15 ICG lymphatic clearance assay

Mice were anaesthetized, and 1 µL (0.5 µg/µL) of Indocyanine green (ICG, Sigma‐Aldrich) was injected into the gingival mucosa using a Hamilton syringe. Fluorescence was recorded at 60, 150, 240, 330 and 420 different points post‐injection using an IVIS Spectrum (PerkinElmer). Mice were allowed to move freely between measurements. In order to measure lymphatic drainage, ICG signal intensity levels were compared between time points and 60‐minute scans.

### 4.16 Detection of BRONJ mice lymphatic drainage function

Cultured macrophages were treated with LPS (10 µg/mL) for 24 h to induce M1 polarization. M1 macrophages were labelled with Dil and injected into the buccal mucosa of mice. Submandibular lymph nodes were excised after 24 h for fluorescence staining to observe macrophage reflux.

### 4.17 Western blot

Lysates of lymphatic endothelial cells were prepared using RIPA buffer. Total protein was separated by polyacrylamide gel electrophoresis and transferred onto nitrocellulose membranes. Primary antibody incubation was carried out overnight at 4°C. Incubation with secondary antibodies conjugated to horseradish peroxidase followed by enhanced chemiluminescence detection was carried out the following day. Antibodies are listed in Supporting Information.

### 4.18 Statistical analysis

All experiments were conducted independently at least three times. Data for each group are shown as mean ± standard deviation. GraphPad Prism 9 software was utilized for the statistical analyses. The paired Student's t‐test was used to compare the two groups. One‐way analysis of variance, followed by Dunnett's post hoc test for multiple comparisons, was used to assess differences among multiple groups. We considered a *p*‐value of .05 to be statistically significant.

## AUTHOR CONTRIBUTIONS

Hongbing Jiang designed and organized experiments. Rongyao Xu and Jiang H supervised the experiments and edited the manuscript. Ziyue Qin and Hanyu Xie performed the experiments, analyzed data and wrote the manuscript. Pengcheng Su and Zesheng Song conducted data acquisition and prepared figures. Songsong Guo, Yu Fu and Ping Zhang provided critical samples and assisted with editing a revised version. All authors read and approved the final manuscript.

## CONFLICT OF INTEREST STATEMENT

The authors declare no conflict of interest.

## ETHICS STATEMENT

All animal experiments were conducted in accordance with the guidelines of the Animal Care Committee of the School of Stomatology, Nanjing Medical University, and received approval from the Ethics Committee (Approval No. IACUC‐1805006 and IACUC‐2308037).

## Supporting information



Supporting Information

Supporting Information

Supporting Information

Supporting Information

Supporting Information

## Data Availability

All data and materials are available.
