# Supplementary material for: Targeting endoplasmic reticulum stress‐induced lymphatic dysfunction for mitigating bisphosphonate‐related osteonecrosis
Source: Clin Transl Med. 2024 Nov 9;14(11):e70082. doi: 10.1002/ctm2.70082 (PMC11550091; doi:10.1002/ctm2.70082)
Supplement: Supplementary file 1 — Supporting Information [file CTM2-14-e70082-s001.docx]

**Supplementary Materials 1**

**
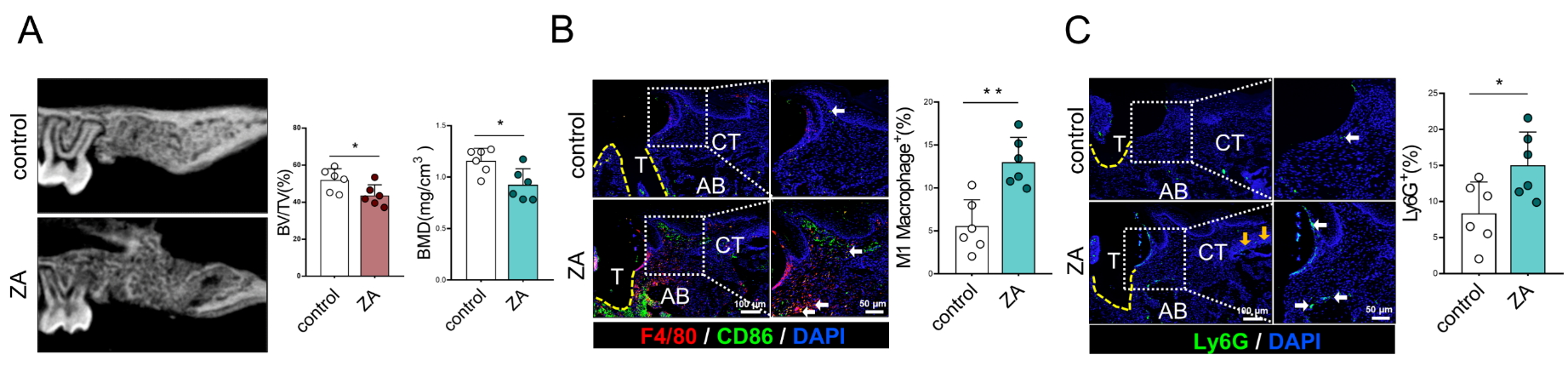
**

**Figure S1.** BRONJ mice exhibit impaired bone healing in tooth extraction socket, with accumulated inflammation. (A) Micro-CT analysis of tooth extraction socket in mice; (B) Immunofluorescence staining of M1 macrophages and (C) neutrophils in tooth extraction socket of mice. A total of 6 subjects were analyzed. The results are presented as the mean ± standard deviation. * *p* < 0.05; ** *p* < 0.01.


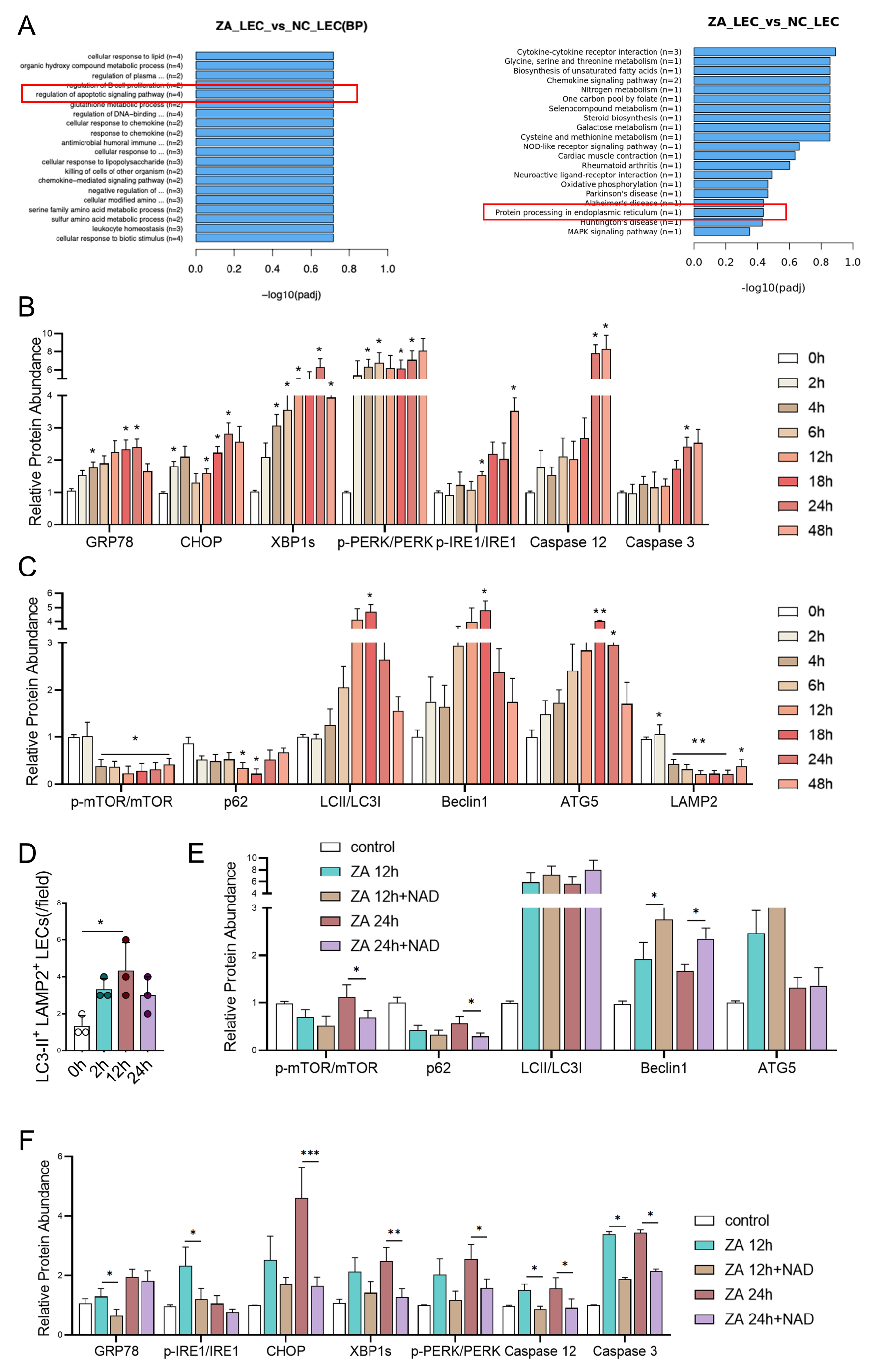


**Figure S2.** ZA induces ERS-apoptosis in LECs. (A) RNA-seq analysis of ZA-treated LECs. Quantification of WB analysis of Figure 2C (B) and Figure 2D (C) plotted relative to the control group. (D) Quantitative fluorescence analysis of autolysosomes in LECs at different time points after ZA treatment. (E) Bar graph represented semi-quantification of autophagy-associated proteins in Figure 2G. (F) Bar graph represented semi-quantification of ERS-associated proteins in Figure 2H.A total of 3 subjects were analyzed. The results are presented as the mean ± standard deviation. * p < 0.05; ** p < 0.01; *** p < 0.001.


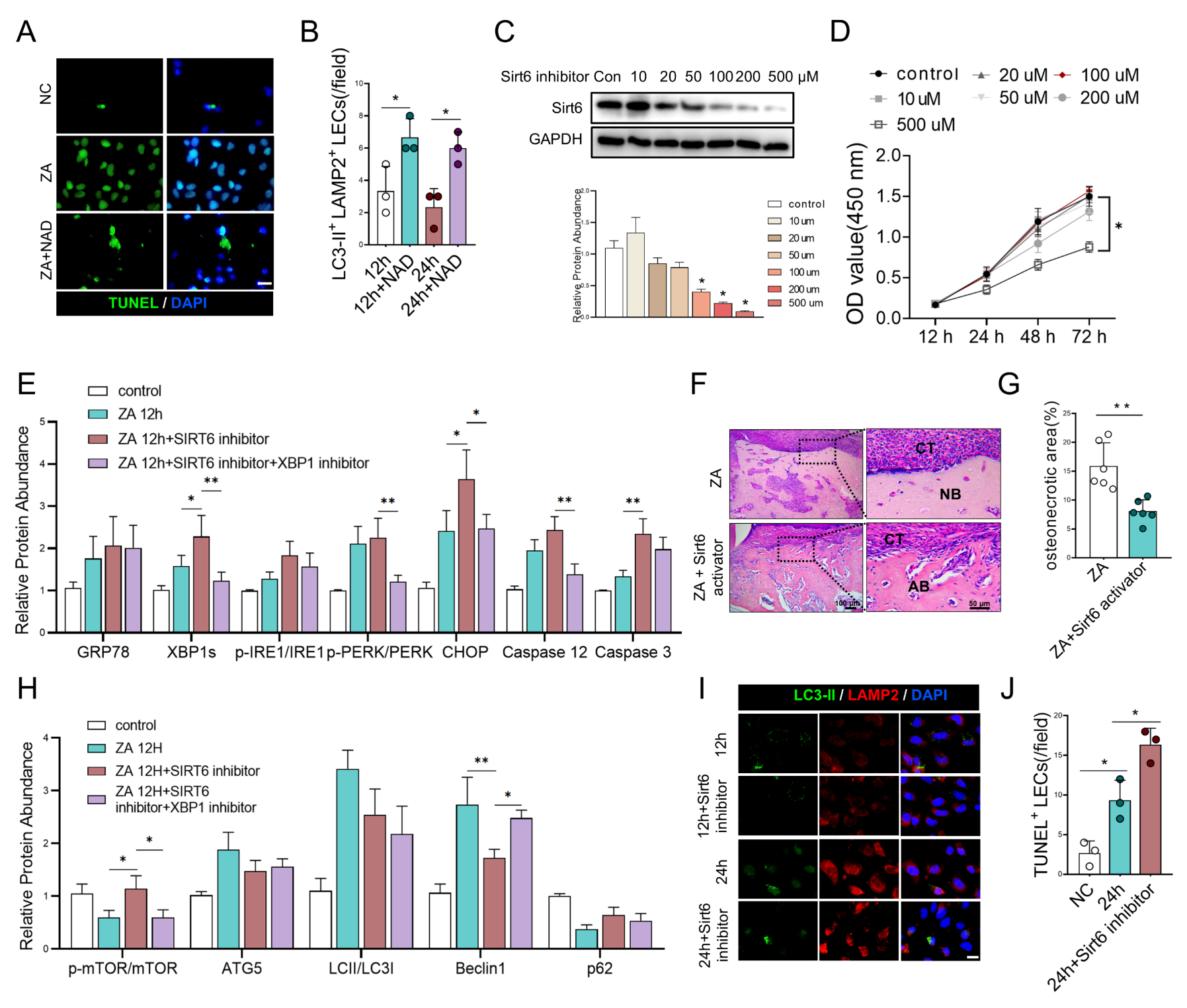


**Figure S3.** Sirt6 mediates ERS-apoptosis in LECs induced by ZA. (A) TUNEL staining to detect apoptotic LECs after ZA and NAD^+^ treatment. (B) Quantitative fluorescence analysis of autolysosomes in LECs from groups treated with ZA and NAD^+^. (C) The effect of Sirt6 inhibitor on Sirt6 protein expression at different concentrations detected by WB. (D) CCK-8 assay to evaluate the effect of different concentrations of Sirt6 inhibitors on cell viability. (E) Quantitative analyses of the protein levels revealed by WB in Figure 2K. (F) HE staining revealed the necrotic bone area and quantitative analysis (G) in BRONJ mice. (H) Quantitative analyses of the protein levels revealed by WB in Figure 2L. (I) Immunofluorescence detection of autolysosomes expression in LECs treated with ZA and SIRT6 inhibitor. (J) Quantitative Analysis of TUNEL staining in LECs after treated with ZA and SIRT6 inhibitor in Figure 2M. A total of 3 subjects were analyzed. The results are presented as the mean ± standard deviation. * p < 0.05; ** p < 0.01.


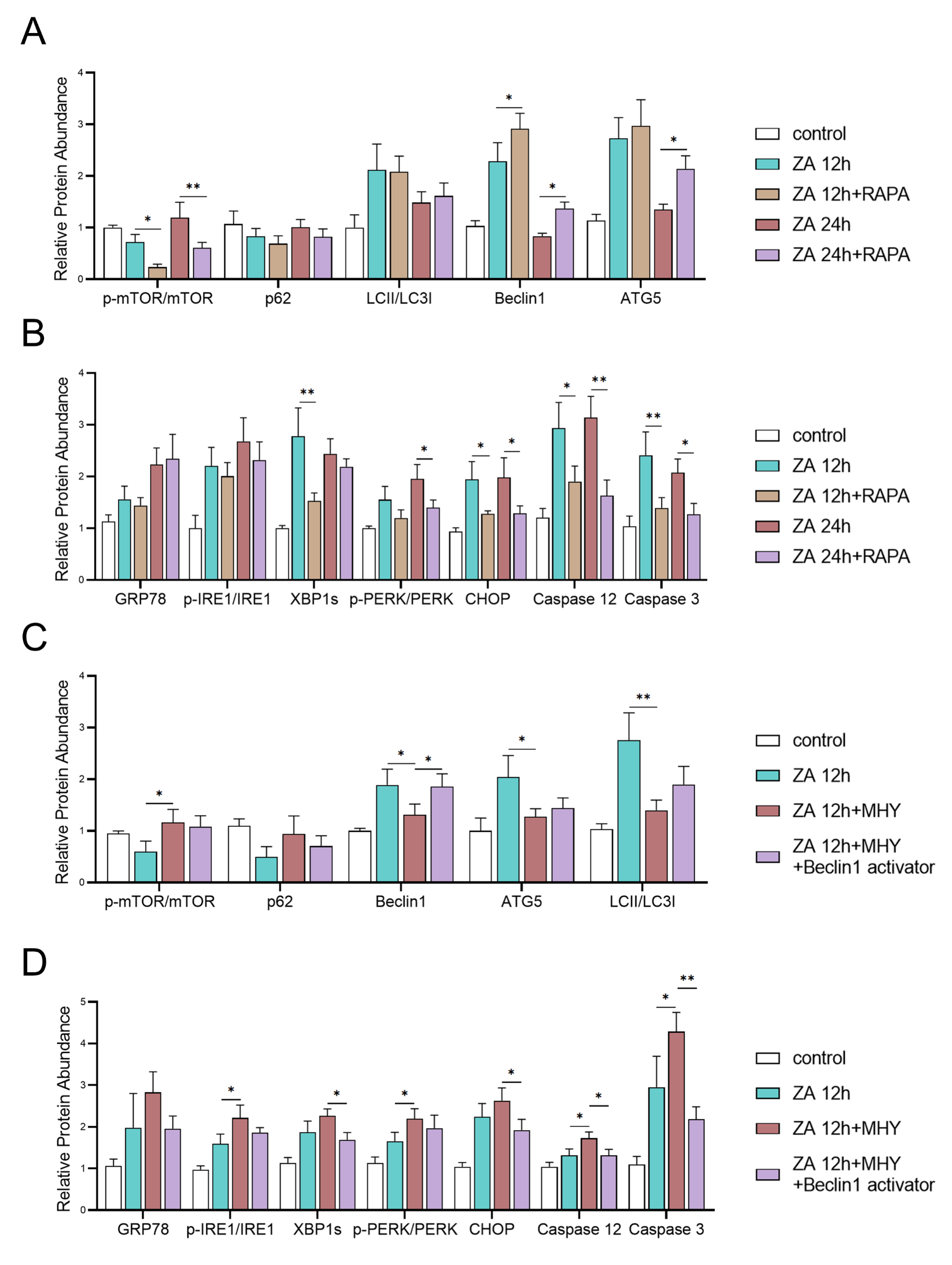


**Figure S4.** Autophagy modulates ERS-apoptosis in LECs induced by ZA. Quantitative analysis of the effect of RAPA on autophagy (A) and ERS-related protein (B) expression in ZA-treated LECs. Quantitative analysis of the effects of two autophagy modulators on autophagy (C) and ERS-related protein (D) expression in ZA-treated LECs. A total of 3 subjects were analyzed. The results are presented as the mean ± standard deviation. * p < 0.05; ** p < 0.01.


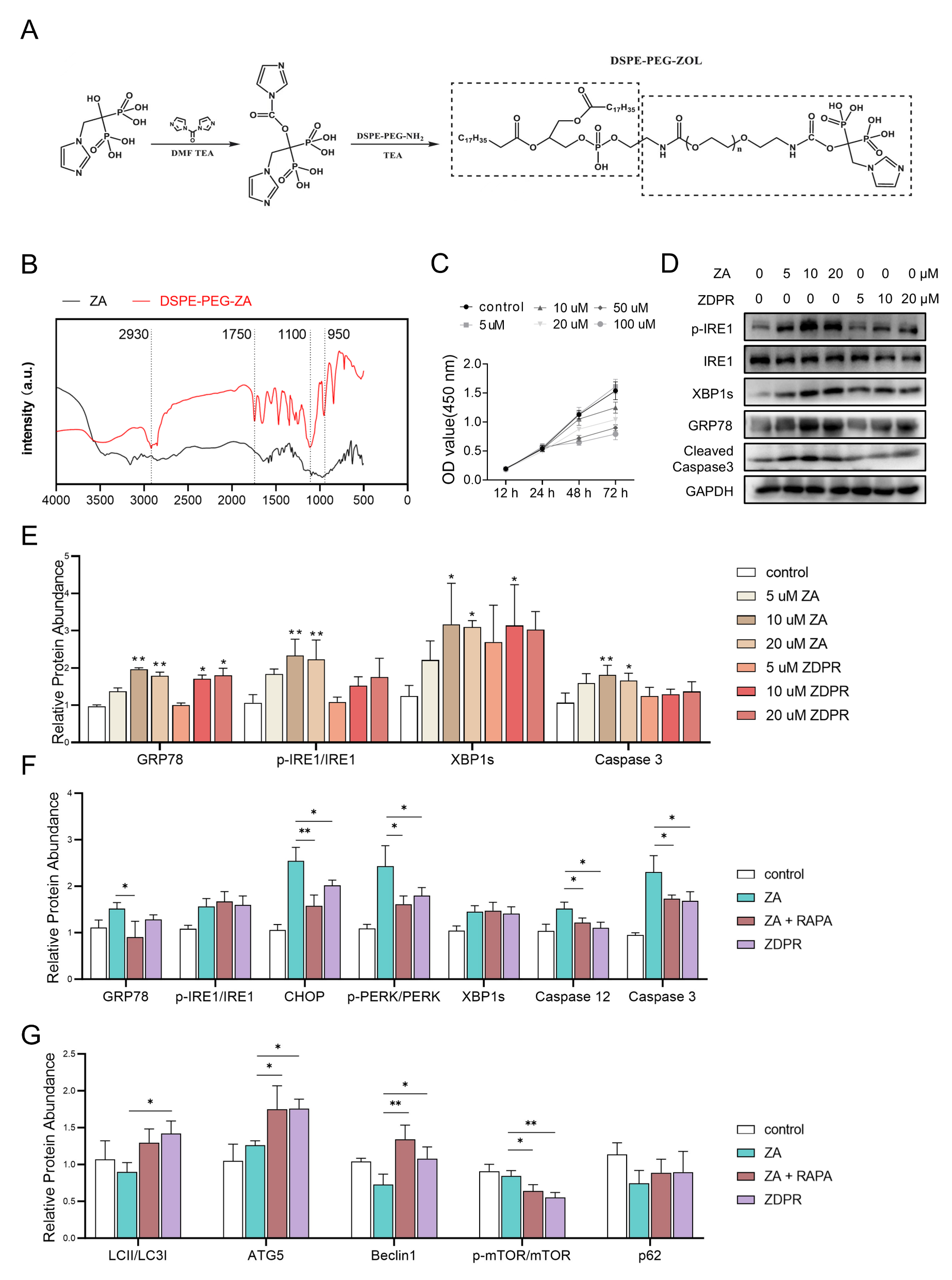


**Figure S5.** ZDPR alleviates ERS-apoptosis in LECs compared to ZA treatment. (A) Schematic representation of the synthesis of the ZDPR. (B) The *FTIR* spectra of samples ZA and ZDPR. (C) Cell viability was measured by *CCK8* staining in groups treated with ZDPR at different concentrations. (D) WB analysis of ERS-related protein expression and quantitative assessment (E) in ZDPR-treated LECs at different concentrations. (F) Quantitative analysis of the effects of ZDPR and RAPA on ERS-related protein in Figure 6F. (G) Quantitative analysis of the effects of ZDPR and RAPA on autophagy-related protein in Figure 6G.A total of 3 subjects were analyzed. The results are presented as the mean ± standard deviation. * p < 0.05; ** p < 0.01.


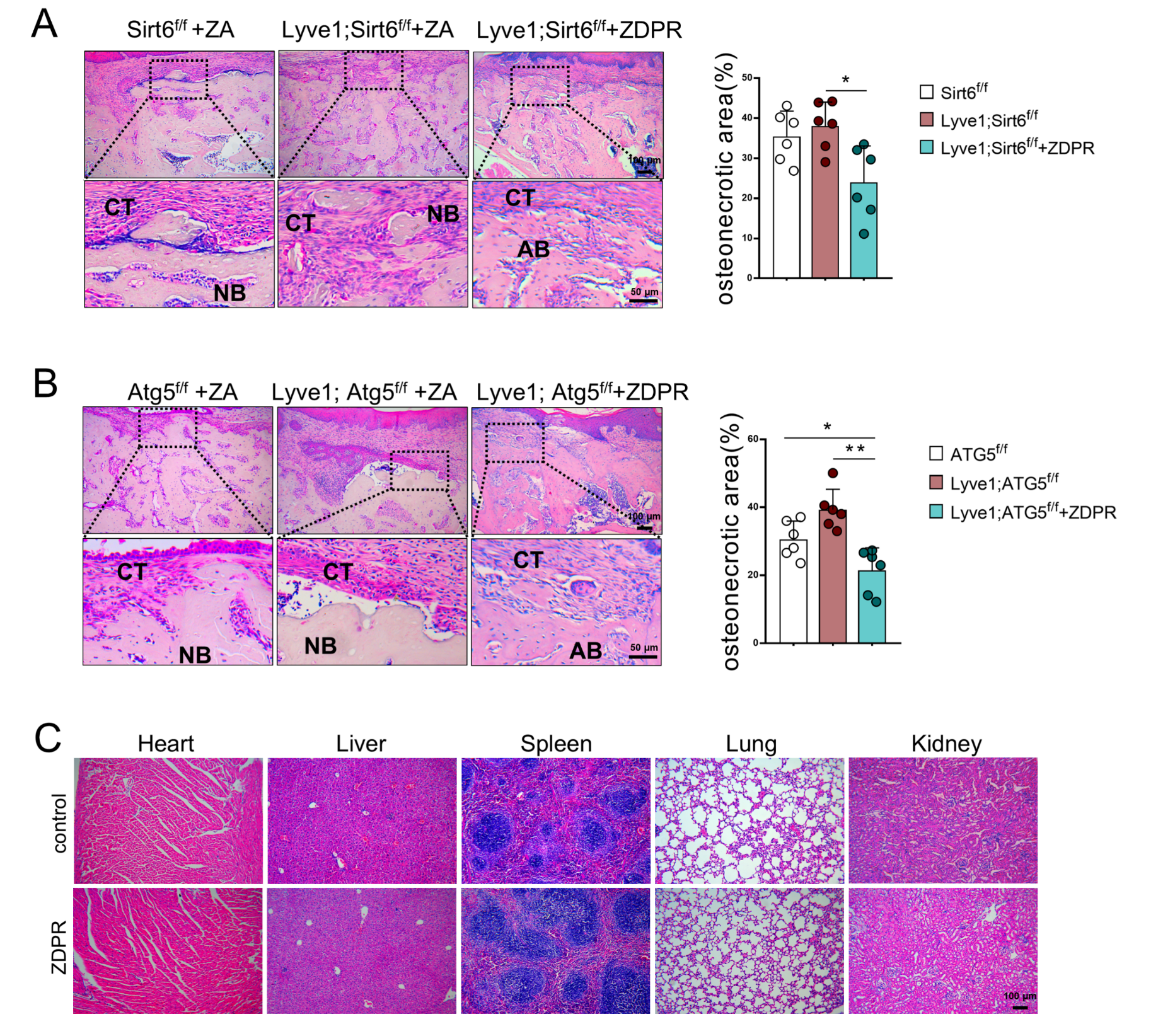


**Figure S6.** ZDPR alleviates BRONJ in *Lyve1^creERT^; SIRT6^fl/fl^* and *Lyve1^creERT^; ATG5^fl/fl^* mice compared to ZA treatment. (A&B) HE staining revealed bone healing in tooth extraction and quantification of necrotic bone area across different treatment groups. (C) Histological evaluation of different organs (*heart*, *liver*, *spleen*, *lung*, *and* *kidney*) from control and ZDPR-treated mice. A total of 6 subjects were analyzed. The results are presented as the mean ± standard deviation. * p < 0.05; ** p < 0.01.
